# Exploration of stem endophytic communities revealed developmental stage as one of the drivers of fungal endophytic community assemblages in two Amazonian hardwood genera

**DOI:** 10.1038/s41598-019-48943-2

**Published:** 2019-09-03

**Authors:** Demetra N. Skaltsas, Fernanda Badotti, Aline Bruna Martins Vaz, Felipe Ferreira da Silva, Romina Gazis, Kenneth Wurdack, Lisa Castlebury, Aristóteles Góes-Neto, Priscila Chaverri

**Affiliations:** 1 University of Maryland, Department of Plant Science and Landscape Architecture, 2112 Plant Sciences Building, College Park, Maryland, 20742 USA; 2 U.S. Department of Agriculture, Agricultural Research Service, Mycology and Nematology Genetic Diversity and Biology Laboratory, 10300 Baltimore Avenue, Beltsville, Maryland 20705 USA; 3 Oak Ridge Institute for Science and Education, ARS Research Participation Program, MC-100-44, Oak Ridge, TN 37831 USA; 4 Centro Federal de Educação Tecnológica de Minas Gerais, Departamento de Química, 30421-169, Belo Horizonte, Minas Gerais 30421-169 Brazil; 5 Universidade Federal de Minas Gerais, Departamento de Microbiologia, 31270-901, Belo Horizonte, Minas Gerais 31270-901 Brazil; 6 University of Florida, Department of Plant Pathology, Tropical Research & Education Center, 18905 SW 280 Street, Homestead, Florida 33031 USA; 7 Smithsonian Institution, Department of Botany, National Museum of Natural History, P.O. Box 37012, Washington, District of Columbia 20013 USA; 8 Escuela de Biología, Centro de Investigaciones en Productos Naturales, Universidad de Costa Rica, San Pedro, San José, 11501 Costa Rica USA

**Keywords:** Tropical ecology, Plant symbiosis

## Abstract

Many aspects of the dynamics of tropical fungal endophyte communities are poorly known, including the influence of host taxonomy, host life stage, host defence, and host geographical distance on community assembly and composition. Recent fungal endophyte research has focused on *Hevea brasiliensis* due to its global importance as the main source of natural rubber. However, almost no data exist on the fungal community harboured within other *Hevea* species or its sister genus *Micrandra*. In this study, we expanded sampling to include four additional *Hevea* spp. and two *Micrandra* spp., as well as two host developmental stages. Through culture-dependent and -independent (metagenomic) approaches, a total of 381 seedlings and 144 adults distributed across three remote areas within the Peruvian Amazon were sampled. Results from both sampling methodologies indicate that host developmental stage had a greater influence in community assemblage than host taxonomy or locality. Based on FunGuild ecological guild assignments, saprotrophic and mycotrophic endophytes were more frequent in adults, while plant pathogens were dominant in seedlings. *Trichoderma* was the most abundant genus recovered from adult trees while *Diaporthe* prevailed in seedlings. Potential explanations for that disparity of abundance are discussed in relation to plant physiological traits and community ecology hypotheses.

## Introduction

Fungal endophytes are found in every plant species and tissue type^1^, and have been the subject of numerous research studies. Nevertheless, information regarding turnover of fungal endophytes from one developmental stage of a plant to another (i.e., from seed to seedling to adult) is limited. Characterizing communities at different developmental stages can shed light onto community dynamics and provide clues on how these highly complex communities are assembled and how they change through time. The few available studies on developmental stages have focused on endophytes isolated from seeds and/or emerging seedlings, or compared older to newly flushed leaves on a single adult plant^2^. Others have involved the inoculation of seedlings with fungal endophytes recovered from taxonomically unrelated adult plants with the objective to assess the effect on plant growth^3,4^, disease resistance^5^, or tolerance to abiotic stress^6^.

Studies comparing the fungal endophytic communities of adults to those of their offspring have focused on leaf tissue^7–10^. In adults, tree diameter has been used to gain insight into the relationship between host age and fungal endophyte richness and abundance. Although these studies have yielded conflicting results, tree age has been found to influence fungal endophyte richness^11,12^. Few studies have evaluated the fungal community harboured within inner bark tissue (phloem and cambium) of tropical hardwood species^13–15^. Communities living within the trunk/stem are thought to be less stable than the ones inhabiting leaves, and these communities are expected to vary with host age^16^. In the tropics, where plant diversity is high and species density is low, host specificity among endophytes has rarely been reported^17,18^, although several studies have shown that host identity does influence endophytic communities^19–21^.

In this study, the endophytic communities of four species of *Hevea* and two species of *Micrandra* (Euphorbiaceae) distributed in remote and underexplored areas of the Peruvian Amazon were characterized. Species in both genera produce copious latex, which is tapped in *Hevea brasiliensis* for producing commercial natural rubber. This study had three main objectives: 1) determine the influence of host identity on community species composition and abundance in adult and seedlings; 2) determine the influence of geographical distance on community species composition and abundance in adult and seedlings; and 3) investigate patterns observed between adult and seedlings when using culture-dependent versus culture-independent approaches.

## Results

### Endophyte isolates

A total of 2,061 endophytic fungal isolates were obtained from 525 individuals belonging to the four species of *Hevea* (*H*. *brasiliensis* [HEBR], *H*. *guianensis* [HEGU], *H*. *nitida* [HENI], *H*. *pauciflora* [HEPA]) and two species of *Micrandra* (*M. elata*, [MIEL], *M*. *spruceana* [MISP]). Stem and inner bark tissue were obtained from individuals at two developmental stages (seedling and adult trees) from three distinct geographic localities in the Peruvian Amazon (Amazon Conservatory for Tropical Studies Biological Station [NAPO], Allpahuayo-Mishana National Reserve [ALPE], and Jenaro Herrera Research Center [JEHE]). The recovery rate differed between the two developmental stages across all three locations. From seedlings, 1,237 isolates were recovered (83% average recovery rate), and 824 from adults (66% average recovery rate) (Table 1).

**Table 1:** Summary of locations, host tree species and the number of trees and developmental stages sampled from Amazon Conservatory for Tropical Studies Biological Station (NAPO), Allpahuayo-Mishana National Reserve (ALPE), and Jenaro Herrera Research Center (JEHE), as well as the total number of endophytes recovered per tree species, location and developmental stage.

Site	Host Tree Species	No. of Adult trees that had seedlings	No. of seedlings sampled (Stem)/No. subsamples/No. of subsamples with endos	No. of adult trees sampled (inner bark/No. subsamples/No. of subsamples with endos	No. of endos recovered		Recovery Rates (%)	
					Seedling	Adult	Seedling	Adult
ALPE	*Hevea guianensis*	16	48/144/127	16/81/53	180	69	88%	65%
	*Hevea pauciflora*	15	45/135/103	16/117/70	132	81	76%	60%
	*Micrandra elata*	10	30/63/38	15/126/84	54	92	60%	67%
	*Micrandra spruceana*	5	15/45/41	9/54/45	58	52	91%	83%
	Total sampled	46	141/396/309	55/387/256	424	294	78%	66%
JEHE	*Hevea brasiliensis*	13	39/126/105	13/54/23	121	51	83%	43%
	*Hevea nitida*	10	30/99/80	10/45/27	94	30	81%	60%
	*Micrandra spruceana*	14	42/126/112	15/108/70	181	74	89%	65%
	Total sampled	37	114/351/297	38/207/120	396	155	84%	58%
NAPO	*Hevea guianensis*	16	48/144/133	21/189/153	197	201	92%	81%
	*Hevea nitida*	9	27/72/54	11/72/45	64	58	75%	63%
	*Micrandra spruceana*	17	51/144/121	19/162/99	156	116	84%	61%
	Total sampled	42	126/360/308	51/423/297	417	375	86%	70%
Totals	Total sampled	125	381/1107/914	144/1017/673	1237	824	83%	66%

**Figure 1:**
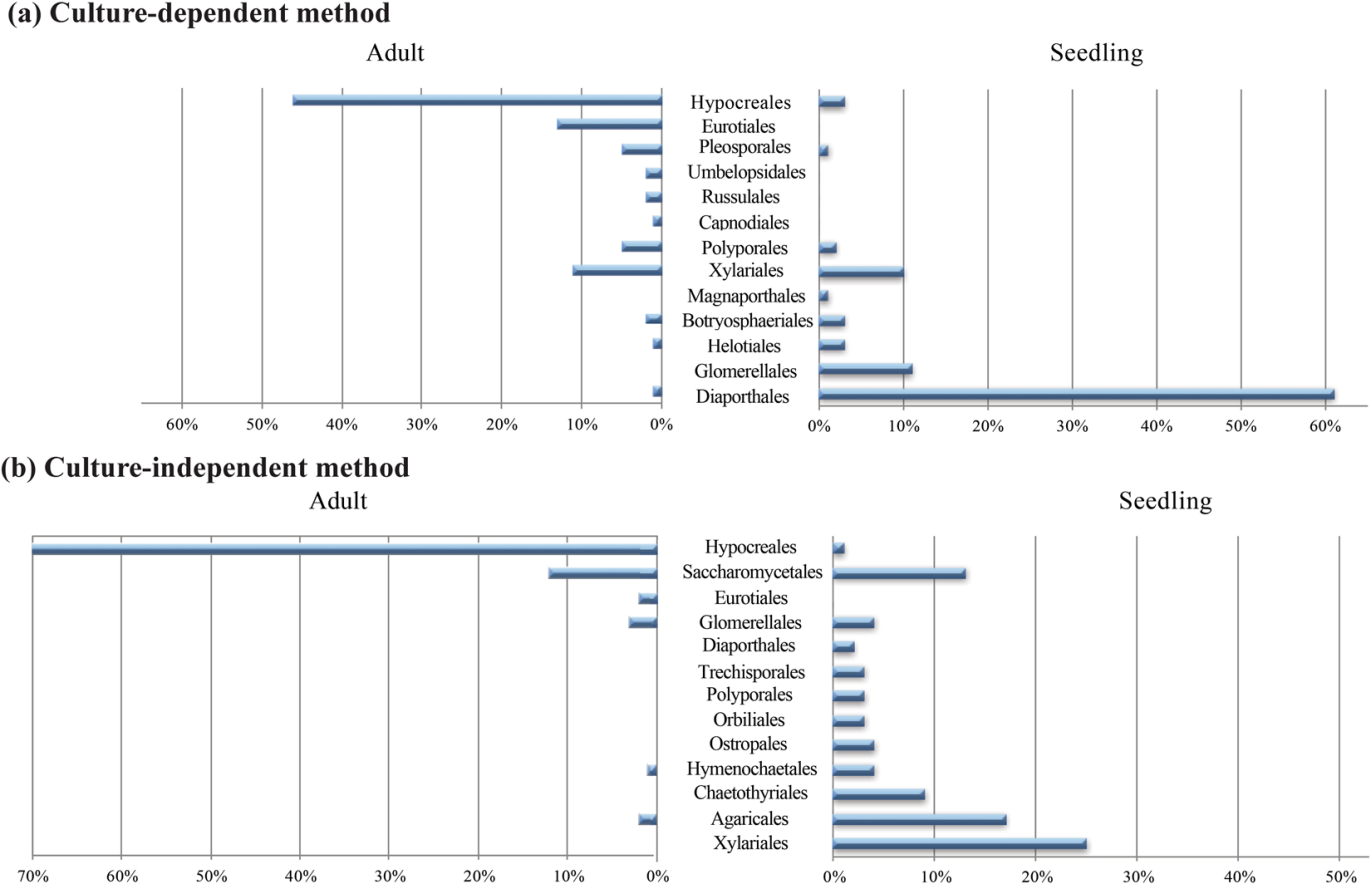
Relative abundance of orders present in each developmental stage under the two different sampling approaches; (**a**) The most abundant taxonomic orders recovered from seedling stems and adult inner bark using culture-dependent method, (**b**) The taxa with the highest incidence frequency captured using culture-independent method.

A total of 356 putative fungal species (OTUs) belonging to 136 genera were recovered. Endophyte communities from adult trees were composed of 255 species and seedling communities were composed of 153 species, with 52 species recovered from both. A total of 18% of the putative species were resolved at the genus level, while 82% could only be classified at higher taxonomic ranks (Supplementary Table S1). In all three locations for all host species examined, Ascomycota dominated the fungal endophytic community in seedlings (97%) and adults (89%). Basidiomycota incidence was lower in both seedlings (3%) and adults (8%). Mucoromycotina were only isolated from adult tree samples (2.7% of isolates). The most abundant fungal orders for endophytic communities in adult trees were Hypocreales (46%), Eurotiales (13%) and Xylariales (11%) (Fig. 1a). The most abundant orders for communities in seedlings were Diaporthales (61% of isolates), Glomerellales (11%), and Xylariales (10%) (Fig. 1b). Three genera were most prevalent in adult trees: *Trichoderma* (26%), *Penicillium* (9%), and *Tolypocladium* (6%) while all the other genera isolated (142 out of 148) were found at relative abundances below 6%. In seedlings, *Diaporthe* (61%) and *Colletotrichum* (12%) were the two most abundant genera while 86 other genera were isolated in abundances below 3%. *Diaporthe* was the most abundant genus recovered from *Hevea* and *Micrandra* seedlings, except for *M. elata* (sampled only in ALPE), where *Pezicula* (28%) and *Colletotrichum* (26%) dominated.

### Diversity estimates of individual and combined datasets

**Figure 2:**
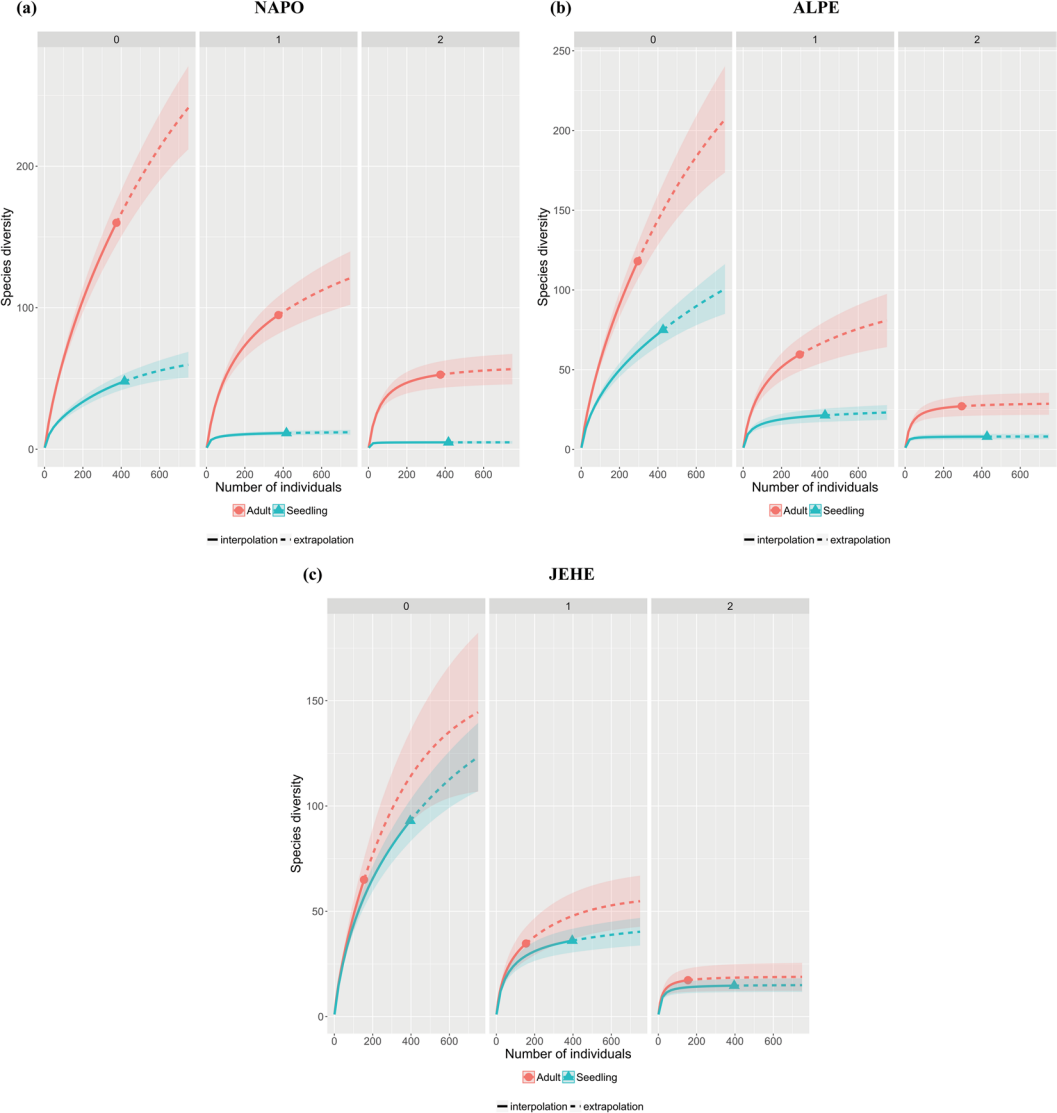
Culture-dependent approach. Species accumulation and diversity curves for endophytes sampled from adult and seedling trees separated by location. (**a**) the Amazon Conservatory for Tropical Studies Biological Station (NAPO), (**b**) Allpahuayo-Mishana National Reserve (ALPE), and (**c**) The Jenaro Herrera Research Center (JEHE). Metrics include richness (q = 0), Shannon HN (q = 1), Simpson’s HN (q = 2).

Richness and diversity for endophytic communities of adult trees was not significantly different within each location, regardless of host taxonomy (Supplementary Table S2). Similarly, richness and diversity for endophytes isolated from seedlings were not significantly different for all hosts within location (Supplementary Table S2). Based on the non-overlap of the 95% CI of the species accumulation curves, richness and diversity was higher in adult trees at individual locations and when locations were combined (Supplementary Table S2, Fig. 2a,b), except for JEHE. In JEHE, fungal richness and diversity for seedlings were greater than for the adult trees (Supplementary Table S2, Fig. 2c).

Species accumulation curves increased steeply and did not reach an asymptote for either seedling or adult tree fungal communities (Supplementary Figs S1–S3). Curves for Shannon HN and Simpson HN diversity reached, or nearly reached, an asymptote for all seedling fungal communities (Figs S2a, S3a and S4a). For adult-tree fungal communities, Simpson HN diversity curves reached, or nearly reached, an asymptote at all three locations. Shannon HN diversity curves; however, did not near or reach asymptote at any location, despite the slopes being less steep than the species accumulation curves (Figs S2b, S3b and S4b). Based on projected diversity^22^, some samples were nearly complete while others were insufficiently sampled (Supplementary Table S2).

### Comparative analyses among host species and across sites, in seedlings and adults

**Figure 3:**
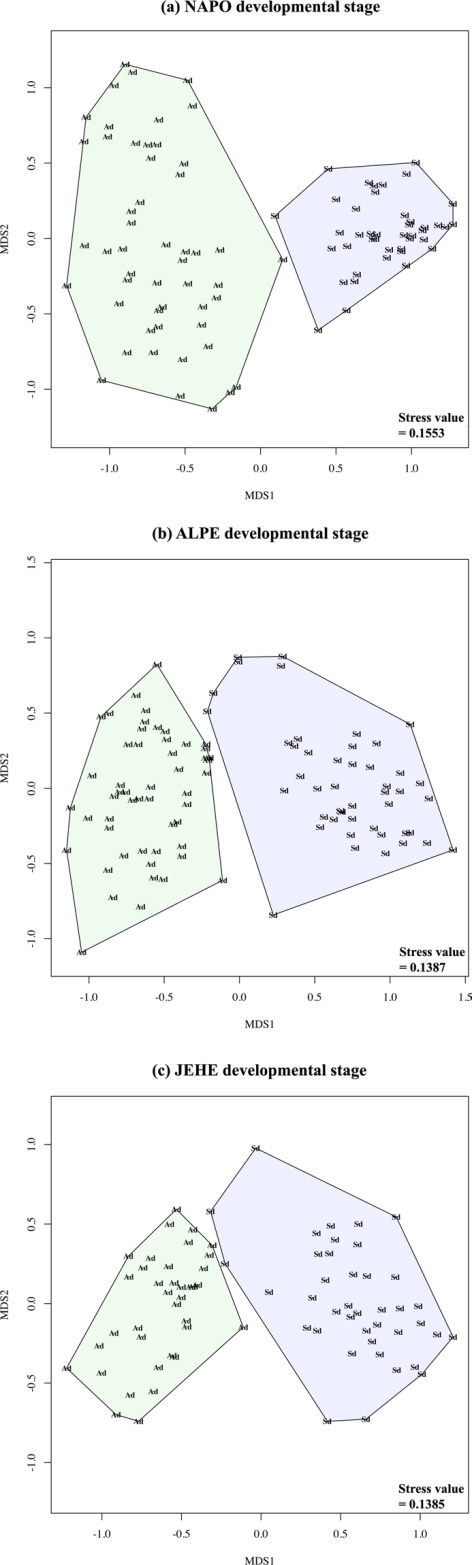
Culture-dependent approach. Nonmetric Multidimensional Scaling (NMDS) analyses using Bray-Curtis distance for quantitative (abundance) data with stress values. Data partitioned by developmental stage (Ad: Adult [green], Sg: Seedling [blue]) within each location (**a**) the Amazon Conservatory for Tropical Studies Biological Station (NAPO), (**b**) Allpahuayo-Mishana National Reserve (ALPE), and (**c**) Jenaro Herrera Research Center (JEHE).

**Figure 4:**
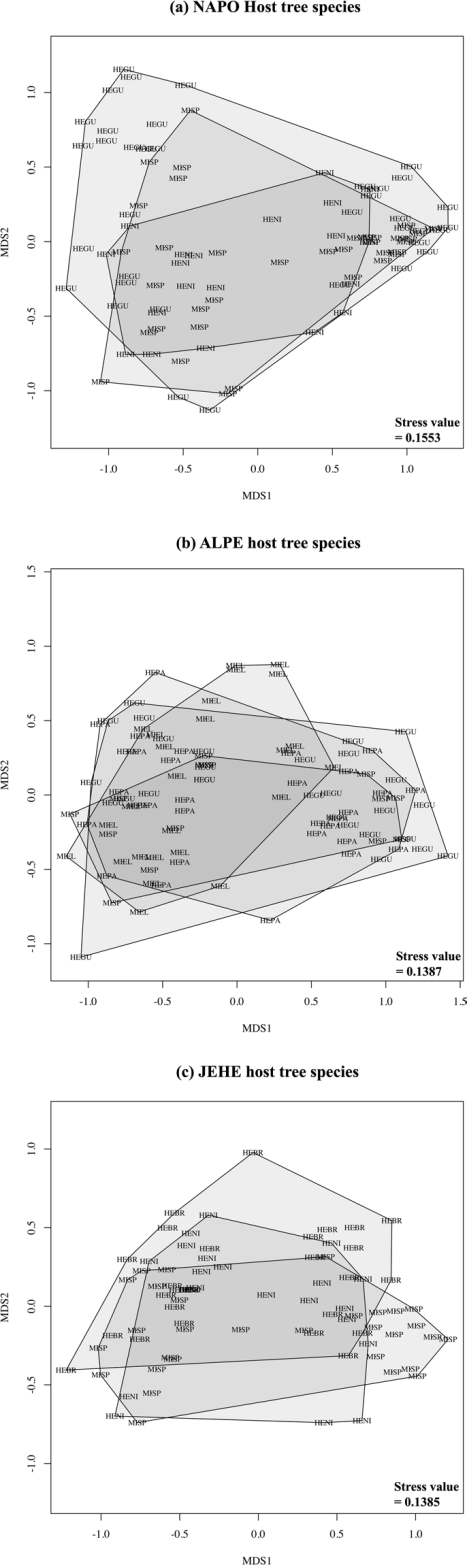
Culture-dependent approach. Nonmetric Multidimensional Scaling (NMDS) analyses using Bray-Curtis for quantitative (abundance) data with stress values. Data partitioned by tree host species (HEBR: *Hevea brasiliensis*, HEGU: *Hevea guianensis*, HENI: *H*e*vea nitida*, HEPA: *H*e*vea pauciflora*, MIEL: *Micrandra elata*, and MISP: *Micrandra spruceana*) and location (**a**) the Amazon Conservatory for Tropical Studies Biological Station (NAPO), (**b**) Allpahuayo-Mishana National Reserve (ALPE), and (**c**) Jenaro Herrera Research Center (JEHE).

Non-metric multidimensional scaling (NMDS) analysis revealed distinctiveness between fungal communities associated with the two developmental stages of host trees (stress = 0.1341) (Supplementary Fig. S4a). This grouping was strongly significant according to ANOSIM (p = 0.001). Seedling and adult trees had different fungal communities across hosts (stress value range 0.0988–0.1484), and across sampling sites (stress value range 0.1385–0.1553, ANOSIM p = 0.001) (Fig. 3, Supplementary Fig. S5). There was no clear distinction between the endophytic communities from different host species when the data was not partitioned by developmental stage within each locality (ANOSIM p > 0.005) (Fig. 4, Supplementary Table S3).

Across all three sampling sites, fungal endophytic community similarity decreased when the geographic distances between the pairwise set of samples increased, except for adult *H. nitida* (Supplementary Table S4). There were very few fungal species that overlapped between seedling and their adult counterparts; overlapped species ranged from two to four, with the exceptions of HEGU in NAPO (6) and MIEL in ALPE (1) (Supplementary Table S5). For adult trees within each location, most species were unique to a host, largely due to the high percentage of singletons and doubletons (Supplementary Fig. S6). The exceptions, where host species had more endophyte species which overlapped between hosts than unique ones, were HEGU and MISP in ALPE, and HENI in NAPO. For seedling trees within each location, the trend was reversed. There were generally more endophyte species that overlapped between hosts than species unique to a single host species.

Core species, those occurring in more than 50% of individuals within a tree species within a site, were only identified from adult HEGU, HEPA and MISP in ALPE, HENI in NAPO, and MISP in JEHE (Supplementary Table S6). No pattern of host preference was observed across the three sites for adults. MISP in ALPE had a different core species (*Neopestalotiopsis* species 3) than MISP in JEHE (*Trichoderma* species 21), while there were no core species recovered from MISP in NAPO (Supplementary Table S6). Seedlings from NAPO had the most fungal species that met core criteria (NAPO 5, ALPE 4, JEHE 3). In ALPE and NAPO, three of the identified core species met the core criteria in multiple hosts, while in JEHE only one core species (*Diaporthe* species 1) met the core criteria for the three host species (Supplementary Table S6).

### Fine scale comparative analyses between adults and seedlings, using culture-independent approaches for one collection site (NAPO)

A total of 1,086,242 reads from 91 trees belonging to three host species (*H. guianensis, H. nitida*, *M. spruceana*) met bioinformatics quality control measures for further analyses. There were 1,039 putative fungal species (OTUs) identified belonging to 436 genera. When considering the number of OTUs, the number of reads, and incidence frequencies, Ascomycota dominated the fungal endophytic community for both seedlings (57% of OTUs, 61% of reads, 62% incidence frequency) and adult trees (73% of OTUs, 87% of reads, 89% incidence frequency). For adult tree samples, the proportion of Basidiomycota comprising fungal communities (13% of sequence reads, 10% incidence frequency) was similar to that observed using culture-based methods, in which 8% of the isolates recovered from NAPO were Basidiomycota. A higher proportion of Basidiomycota were recovered in seedlings using culture-independent methods (32% sequence reads, 37% incidence frequency) than from cultured-based (0.7%). Low incidence of Mucoromycotina was observed using culture-independent methods (0.23% seedling, 0.48% adult) but a higher incidence was observed from cultured samples (3% of isolates, adult).

Fungal genera recovered using culture methods were underrepresented in the culture-independent survey. In seedlings, 50% of recovered genera were represented in the culture-independent dataset, whereas only 23% were represented for adult endophytic communities. For all adult trees hosts in NAPO, four endophytic genera were the most prevalent: *Acremonium* (18% of sequence reads, 10% incidence frequency), *Debaryomyces* (14%, 10%), *Tolypocladium* (14%, 9%), *Sarocladium* (1%, 8%). *Tolypocladium* was also one of the most abundant genera recovered using culture-dependent methods. No *Sarocladium* species were recovered from cultured samples and less than 1% of recovered isolates were members of *Debaryomyces* and *Acremonium*. *Trichoderma*, the most abundant genus recovered using culture methods, was captured from only one tree sample (HEGU), at a very low abundance (2 reads). For all seedling species in NAPO the two most prevalent genera, *Debaryomyces* and Tricholomataceae species 1, were not recovered using culture methods. *Diaporthe*, the most abundant genus recovered using culture methods, was recovered in 26% of seedling trees at a low abundance (0.14% reads) and incidence frequency (0.61%). *Colletotrichum*, the second most abundant genus recovered using cultured-based methods, was recovered in 43% of seeding trees, also at low abundance (0.56% reads) and incidence frequency (3.60%).

Richness and diversity of endophytic communities recovered using culture-independent methods were significantly different among adults, based on Shannon HN and Simpson HN indices, whereas there were no significant differences observed using culture-dependent methods. HEGU had the greatest richness and diversity, followed by HENI, and MISP (Supplementary Table S7). Endophytic communities from HEGU and HENI seedlings had similar richness and diversity, however, MISP was significantly more diverse. As with culture-dependent method, richness and diversity of endophytic communities in adult trees was greater than in seedlings, except for MISP (Supplementary Fig. S7). The number of species recovered per MISP seedling ranged from 33 to 111 (mean 56, median 50), while the number of species recovered from HEGU and HENI seedlings ranged from 8 to 24 (mean 15, median 13).

Species accumulation curves increased steeply and did not reach an asymptote for either seedlings or adults (Supplementary Fig. S8). Curves for Shannon HN and Simpson HN diversity reached or nearly reached asymptote for HEGU and HENI seedlings while the curve for MISP seedlings remained steep (Supplementary Fig. S8a). For all adult tree species, Simpson diversity curves approached asymptote. Shannon HN diversity curves, however, did not near or reach asymptote, despite the slopes being less steep than the species accumulation curves.

**Figure 5:**
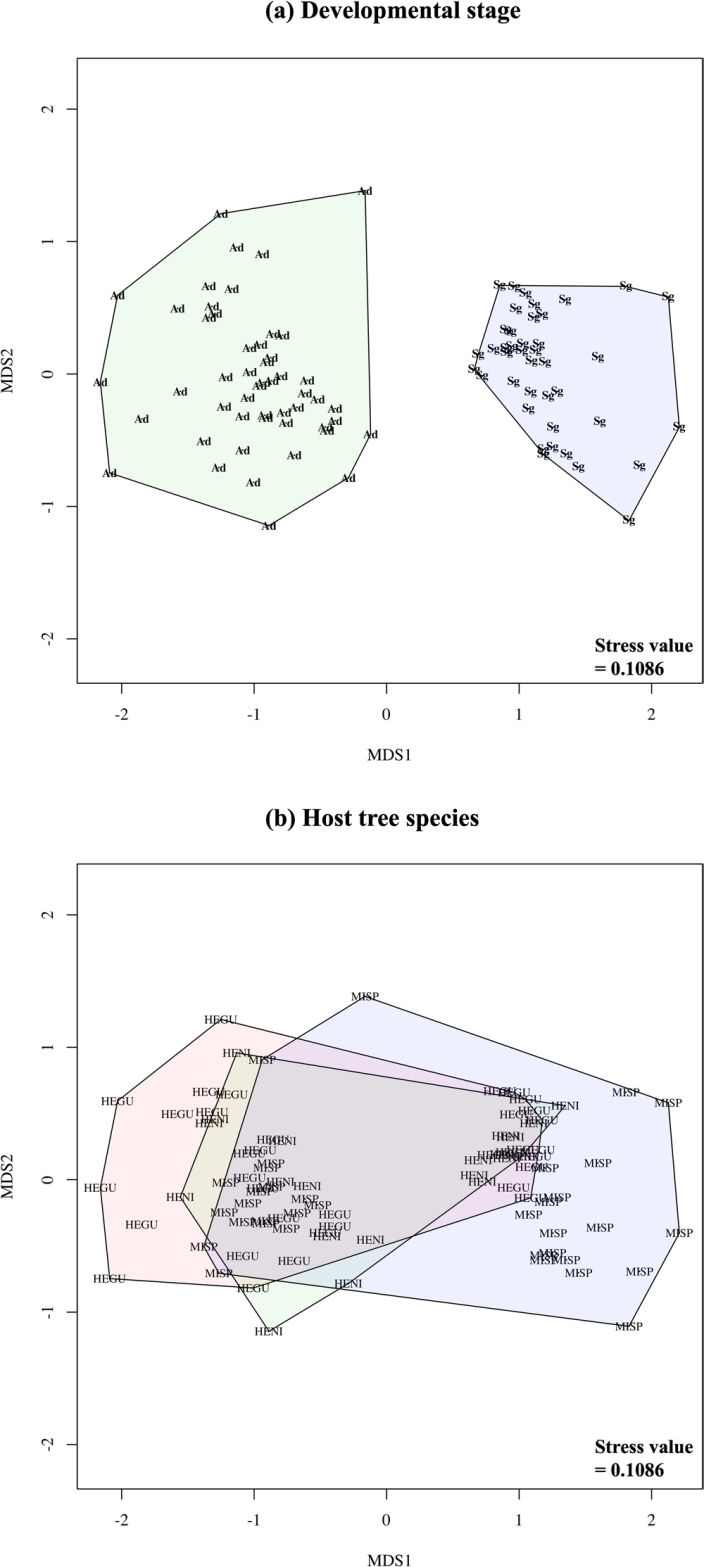
Community similarity results from culture-independent approach. Nonmetric Multidimensional Scaling (NMDS) analyses using Bray-Curtis distance for incidence frequency data for all trees sampled from the Amazon Conservatory for Tropical Studies Biological Station (NAPO). Data partitioned by (**a**) developmental stage (Ad: Adult [green], Sg: Seedling [blue]), and (**b**) tree species (HEGU: *Hevea guianensis*, HENI: *H*e*vea nitida*, and MISP: *Micrandra spruceana*).

Non-metric multidimensional scaling (NMDS) analysis revealed distinctiveness between fungal communities associated with the two developmental stages of host trees (Fig. 5a). Clear separation between the endophytes recovered from seedlings and adult tree communities was observed when either presence/absence data (stress = 0.1086, ANOSIM p = 0.001), or sequence read data (stress = 0.1659, ANOSIM p = 0.001) was analyzed (Supplementary Table S3). There was no significant distinction between the different host species (Fig. 5b). Core species were identified from all adult and seedling host species. As with culture-dependent results, seedling endophytic communities had the most fungal species that met core criteria (16 species), six of the species identified met the core criteria in all three seedling host species. In adult endophytic communities, seven species were identified as core species, four of which met the criteria in all three host species (Supplementary Table S8).

### Ecological guilds

**Figure 6:**
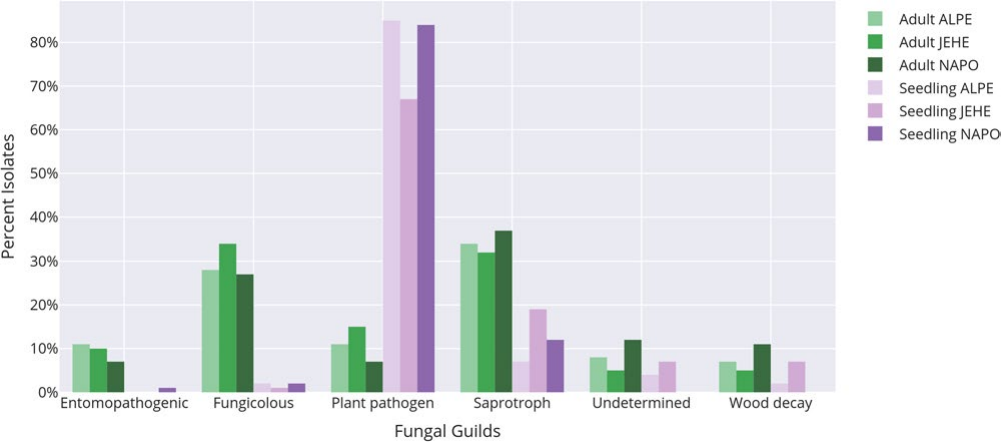
Distribution of function guilds separated by developmental stage and location (Amazon Conservatory for Tropical Studies Biological Station [NAPO], Allpahuayo-Mishana National Reserve [ALPE], and Jenaro Herrera Research Center [JEHE]).

The ecological guilds, based on FunGuild assignment, differed greatly between seedling and adult tree endophytic communities in both culture-dependent and -independent approaches. For culturable endophytes, OTUs in the plant pathogen guild were isolated in a higher percentage from seedlings (79%) than from adult trees (10%) (Fig. 6). Less than 3% of guilds that would be considered beneficial to plant hosts, entomopathogenic (0.24%) and fungicolous (2%), were isolated from seedlings. A higher percentage of endophytes in saprotrophic (35%), fungicolous (29%), entomopathogenic (9%), undetermined (9%) and wood decay (8%) guilds were isolated from adult trees. In addition, the distribution of ecological guilds differed in JEHE. Adults trees in this region harboured more potentially plant pathogenic fungi (JEHE 15%, ALPE 11%, NAPO 7%) and less saprotrophic fungi than what was found in ALPE and NAPO (JEHE 32%, ALPE 34%, NAPO 37%). In seedlings, the overwhelming majority of fungi recovered were members belonging to potentially plant pathogenic taxa. However, in JEHE there were less isolates under this guild recovered (JEHE 67%, ALPE 85%, NAPO 84%) as well as less fungicolous fungi (JEHE 0.5%, ALPE 2%, NAPO 2%).

As with culture-dependent methods, the ecological guilds in culture-independent metabarcoding differed greatly between seedling and adult trees; however, the proportions of fungal guilds comprising the endophytic communities differed between the two methods (Supplementary Table S9). A higher percentage of endophytes in entomopathogenic guild (23% incidence frequency, 30% of sequence reads) and a lower percentage of fungicolous guild (10% incidence frequency, 19% of sequence reads) were captured from adult tree communities than from seedlings. However, endophytes in the plant pathogen guild were similarly recovered from seedling (13% incidence frequency, 3% of sequence reads) and adult trees (10% incidence frequency, 3% of sequence reads). A higher percentage of endophytes with unknown function guilds (41% incidence frequency, 24% of sequence reads) were detected in seedling communities, than in adult tree communities (18% incidence frequency, 24% of sequence reads). Similar to results from the culture-based approach, seedlings harboured less than 1% of guilds that would be considered beneficial to plant hosts, entomopathogenic (0.53% incidence frequency) and fungicolous (0.08% incidence frequency).

## Discussion

In this study, we examined the influence of host identity, host developmental stage, and geographic distance on fungal endophytic communities of four species of *Hevea* and two species of *Micrandra* across three remote areas of the Peruvian Amazon. This is the first systematic study comparing adult to seedling stem tissue of trees. Prior studies have usually compared fungal communities recovered from adult tree and offspring leaves. Our results suggest that host developmental stage, more than host taxonomy or locality, is a key determinant of community assemblage of tropical endophytes.

Seedling and adult hosts had different fungal endophyte communities, in terms of both abundance and species composition, with greater numbers of endophytic fungi recovered from seedlings (83%) than from adult trees (66%). Higher isolation rates have been reported in younger host tissues in other host systems as well, and isolation rates may be a function of the fungal taxa involved, where those taxa from younger tissues are simply faster and easier to grow, or that they do not require a specific growth medium^7–9,23^. Differential diversity has been reported in younger host tissues in other plant systems and may be attributed to important selective factors likely related to host defences that vary according to host species and life stage^7–9^. Immature tissues in *Hevea* are tender, lack lignin and secondary growth, and have not fully developed defensive biochemical pathways that produce fungitoxic compounds such as the coumarin scopoletin^24^, although chitinases are present and seedlings are lactiferous. Younger tissue also contains higher levels of gaseous hydrogen cyanide (HC), which protect against herbivores but inhibit fungicidal enzymes^25,26^.

Host developmental stage, in all analyses, was the greatest factor influencing endophytic community. Adult tree hosts generally harboured richer and more diverse endophytic communities than seedling hosts. *Trichoderma* and *Penicillium* were the most commonly cultured fungi from adult trees but were rarely isolated from seedling stems (0.2%). The opposite pattern was observed for *Diaporthe* and *Colletotrichum*, which were more abundant in seedling stems than in adult trees. Gazis and Chaverri^11^ likewise found *Trichoderma* species to be the dominant culturable endophytic fungi in wild adult *Hevea* trees across localities. The same study found a negative correlation between the presence of *Trichoderma* spp. and the abundance of potentially pathogenic fungi, especially *Diaporthe*. This pattern of one taxon being isolated more frequently from one host developmental stage has been reported for other tree hosts and tissue types as well, suggesting that host developmental stage is a factor that shapes endophytic communities^7–10^.

Seedlings and adult trees in natural forests are generally exposed to the same surrounding *ex-planta* fungal inocula, and regardless of plant tissue type, fungal endophytes penetrate and infect plant hosts similarly through natural openings (lenticels, stomata), wounds, trichomes^27^ or through direct penetrate on (appressoria and penetration peg)^28^, therefore, several factors likely influence differences between developmental stages. Endophytes live between plant cells and they, as well as necrotrophs, lack haustoria^29,30^, indicating that fungal endophytes might be adapted to particular “microenvironments” (e.g., host cells and tissues)^31^, and that seedlings may be more susceptible to colonization by fungal plant pathogenic groups than adults^26,32^, or the Negative Density Dependence (NDD) effect may be occurring due to an accumulation of pathogenic taxa surrounding the parent tree^33,34^. The mechanisms behind these differences are beyond the scope of this study and our results suggest that this is an area that would benefit from further examination. We do note that for these particular hosts early seedling mortality is extremely high based on the abundance of such seedlings relative to the few large adult trees.

Our observations on the influence of geographic distances and locality on endophyte communities illustrate the complexity of the forces that shape biogeographical patterns^35^. Although short geographical distances did not greatly influence community composition longer distances had a significant effect. However, within and across locations there were host species and developmental stage specific differences. There is no evident explanation for the differences observed, and a closer examination of the evolutionary and ecological mechanisms underlying biogeographical patterns (dispersal, selection, drift, and mutations) is needed.

One site differed from the other two in terms of the influence of location on endophyte diversity, richness and community composition. Adult trees generally had a higher endophytic richness than seedlings, except in JEHE where endophytic richness of adult trees was lower than the richness of seedlings as well as lower than the endophytic richness of adults in the other two locations. The most common genera and species in both other two localities were either absent or not dominant in JEHE. *Diaporthe* species 7 was the most recovered species from seedling stems in all three locations. However, the abundance of this species in JEHE was almost half of that recovered from the other two locations.

From an observational assessment of the three locations, JEHE had the most anthropogenic pressures such as forest fragmentation due to small-scale farming and conversion of forested areas into pastures. Interestingly, the data seems to suggest there may be an anthropogenic impact. The impact of anthropogenic activities on the richness and diversity of fungal communities has been well documented^36–39^. However, a more systemic investigation would be required to confirm if this hypothesis is true.

For the one collection site (NAPO) in which both culture-independent and culture-dependent methods were utilized, results of both methods generally agreed on the clear distinction between the taxonomic community composition and abundance of fungal endophytes of adult and seedlings. However, endophyte taxa identified with culture-dependent and culture-independent methods had very little overlap. Dramatic differences were observed in the abundance of some of the commonly isolated genera when compared with culture-independent methods. This may be related to the ability of certain genera, such as *Trichoderma*, to grow readily on artificial media and overgrow other fungi, or the discrepancy may be due to different inherent biases when using metagenomic methods. Those biases may be factors in the initial amplicon library preparation, differential primer annealing, PCR and sequencing artifacts, and contig assembly^40–44^. Previous reports in other systems have also shown that these two methodological approaches recover different endophytes and have suggested that both approaches are complementary and are needed for a comprehensive exploration of these highly diverse communities^45–47^.

## Conclusion

Our findings suggest that host developmental stage, more than host taxonomy or locality, is a key determinant of community assemblage of tropical endophytes. While the tissue sampled from seedlings included the entire stem cross-section and adult tissue predominantly consisted of phloem and cork cambium, there was a higher diversity found among adult samples and very little overlap between the two developmental stages, suggesting that the differences observed may be due to fungal adaptations to the developmental stage. The few relevant papers comparing endophytic communities from adult plant tissues to that of their offspring involved leaves. Their observations were similar to ours in that a higher isolation rate and lower endophytic diversity were observed in younger plant tissue than in mature tissues, and that the endophytic species composition between the developmental stages differed significantly^7–9^. Our results reflect host species within a single family, and only within two plant genera. Expanding sampling space to include higher taxonomic levels may reveal a stronger host effect, however, endophyte studies from the tropics have shown an overall weak effect of host on endophytic communities^18^.

We observed that, although short geographical distances did not greatly influence community composition, longer distances had a significant effect. Many of the fungal genera recovered from seedling and adult trees using culture-dependent methods were dissimilar from those detected using culture-independent methods, however, the pattern of distinctive endophytic communities between the two developmental stages held true.

Despite extensive sampling, our findings revealed other aspects of tropical fungal endophyte community dynamics that need to be investigated: mechanisms that drive the differences between seedling and adults; the effect of anthropologic disturbances; and patterns revealed if additional locations were sequenced.

## Materials and Methods

### Collection sites

Samples were collected from three localities within the Loreto Region of Peruvian Amazon: (1) Amazon Conservatory for Tropical Studies Biological Station (NAPO; 3°14′57.20″S, 72°54′33.60″W), (2) Allpahuayo-Mishana National Reserve (ALPE; 3°58′1.16″S, 73°25′8.11″W), and (3) Jenaro Herrera Research Center (JEHE; 4°53′54.29″S, 73°38′59.80″W). All three locations are high-terraced lowland forests (103–146 m elevation)^48^. NAPO was the most remote location, ALPE was easily accessible but well preserved, and JEHE had the most anthropogenic pressures such as nearby forest fragmentation due to small-scale farming and conversion of forested areas into pastures.

### Endophyte isolation

Endophytic fungi were isolated from stem tissues of a total of 144 apparently healthy adult trees. Four species of *Hevea* (*H*. *brasiliensis* [HEBR], *H*. *guianensis* [HEGU], *H*. *nitida* [HENI], *H*. *pauciflora* [HEPA]) and two *Micrandra*, (*M. elata*, [MIEL], *M*. *spruceana* [MISP]) were sampled. The number of individual trees sampled, per species and site, varied due to their natural low abundance and scattered distribution (Table 1). Trunk diameter (dbh) was measured at 1.4 meters above the ground (*Hevea* spp.) or 1.4 meters above the swell of buttressed prop roots (*Micrandra* spp.). Trees with dbh of 23–100 cm were targeted for sampling. Vouchers for each host tree were collected and deposited at the Universidad Nacional de la Amazonía Peruana (AMAZ).

Three pieces (4 × 5 mm) of inner bark tissue (wood containing cork, cork cambium, and vascular phloem tissue) were excised as described in Gazis and Chaverri^15^ and transferred to individual 35 mm Petri plates containing BBL™ cornmeal-agar (Sigma-Aldrich, St Louis, Missouri, USA) with 2% dextrose and 1% neomycin-penicillin-streptomycin solution to suppress bacterial growth (CMD+). Each adult tree had this procedure replicated three times, at different points around the trunk circumference, yielding a total of nine subsamples per tree.

Three healthy seedlings within 3 m of the parent tree, from the current season (less than 1 year old), and ranging from 30 to 60 cm in length, were randomly collected per adult tree. From each seedling stem, three segments (5 mm long) that included the entire stem with all the inner primary tissues (cortex and vascular, both phloem and xylem), were excised using sterile surgical blades and surface-sterilized as described by Gazis and Chaverri^15^. Each segment was transferred to an individual Petri plate containing CMD+. This procedure was replicated three times, yielding a total of nine subsamples per seedling.

CMD+ plates were incubated at room temperature and emerging colonies were sub-cultured onto BBL™ potato-dextrose-agar (PDA) and incubated at 25 °C for a minimum of 4 days until pure cultures were obtained. The endophyte recovery rate was calculated by dividing the number of subsamples with endophytes by the total number of subsamples^49^.

### Environmental sample collection for direct sequencing

To investigate whether the recovered endophytic community from adults and seedlings varies when using culture-independent versus culture-dependent approaches, additional samples were collected (as described for the culture-based method) from adults and seedlings of *H. guianensis, H. nitida*, and *M. spruceana* in NAPO. Subsamples from each individual adult or seedling were divided into three cryovials (each containing 3 tissue pieces) along with 500 µL of MoBio Bead Solution Buffer (MoBio Laboratories, Carlsbad, California, USA).

### DNA extraction, PCR and sequencing

For culture-dependent approach, mycelial mats from all fungal isolates were harvested directly from PDA plates and suspended in a microcentrifuge tube containing 60 µL of PrepMan® Ultra Reagent (Applied Biosystems, California, USA). DNA was extracted following the manufacturer’s protocol. The nuclear ribosomal Internal Transcribed Spacer (ITS) region containing ITS1, 5.8S, and ITS2 was amplified and sequenced using the primers ITS5 and ITS4^50^. Amplifications were performed following the conditions in Gazis *et al*.^11^. PCR products were sequenced on an ABI 3130xl Genetic Analyzer (Thermo Fisher Scientific, Waltham, Massachusetts, USA) at USDA (USDA-ARS, Beltsville, Maryland, USA) and at Macrogen USA (Rockville, Maryland, USA).

For culture-independent samples, excised plant tissues were ground in tubes prefilled with garnet sand and a 6-mm zirconium oxide satellite bead (OPS Diagnostics LLC, New Jersey, USA.) using a FastPrep® beadmill (MP Biomedicals, Santa Ana, California, USA.). Each tube was treated to three grinding cycles (speed: 5.0 m/s, time: 43 seconds) or until no visually recognizable fragments remained. Total DNA was extracted using the Qiagen® DNeasy Plant Mini Kit according to the manufacturer’s instructions (Qiagen, Hilden, Germany). DNA amplification of the fungal ITS 2 region used fITS7 and ITS4 primers^50,51^. DNA libraries were constructed by MRDNA (www.mrdnalab.com, Shallowater, Texas, USA) using TruSeq DNA Library Prep Kit for paired-end sequencing read lengths of 150 base pairs, following the manufacturer’s guidelines (Illumina, San Diego, California, USA). DNA libraries were sequenced on an Illumina MiSeq by MRDNA following manufacturer’s guidelines. Forward and reverse sequences of each sample were merged into contigs. For quality control and optimization of downstream analyses, reads with 90% of their bases below Q30 quality score and contigs with lengths smaller than 300 base pairs (bp) were discarded, and sequences larger than 300 bp were truncated to 300 bp^52,53^. Singletons, contigs with abundance equal to one, were removed from the dataset and the UCHIME database was used to retrieve and remove chimeras from the dataset^54^.

### OTU delimitation and classification

For culture-dependent method, full-length ITS sequences (~600 bp) from Sanger sequencing of all fungal isolates were aligned using MAFFT version 7.305^55^ on CIPRES^56^ with default parameters and the adjust direction option. MOTHUR version 1.36.1^57^ was used to cluster sequences into operational taxonomic units (OTUs) using the furthest neighbor method and a 99% similarity criterion^58^. One representative from each cluster was chosen and taxonomically classified according to Vaz *et al*.^59^ (Supplementary Table S1). Hereafter, OTUs are considered putative species.

For the culture-independent method, sequences were 300 bp in length; however, most fungal reference sequences in the NCBI nuclear database correspond to the full ITS region (<550 bp). As this can affect search results^60,61^ when assigning OTUs, the following steps were completed: (1) a preliminary BLAST search and gathered GenBank representative sequences of the matched taxa; (2) a dataset constructed with the 1,835 taxa; (3) NCBI representative sequences, along with sequences from both culture-dependent dataset and culture-independent dataset, aligned using MAFFT with the default parameters in CIPRES; (4) reference sequences truncated to 300 bp; and (5) sequences clustered into OTUs using the furthest neighbor method in MOTHUR. The genus limit for the environmental OTUS was the percent similarity at which all the GenBank representative sequences clustered together^59^.

### Diversity estimates of individual and combined datasets

Three orders of Hill numbers (HN) were used to interpolate and extrapolate species richness and diversity for all endophyte communities at tree host level within each locality (species richness, q = 0), common species (Shannon’s entropy, q = 1) and dominant species (inverse Simpson’s, q = 2), decreasingly sensitive to rare species^62,63^. Chao1 was calculated to extrapolate asymptotic richness using iNext version 2.0.14. Hill numbers are expressed as the number of equally abundant species that would be needed to return the same value given by a diversity measure^62,63^. Accumulation and diversity curves were built using iNext version 2.0.14. A 95% confidence interval was generated by applying 1,000 bootstrap iterations. Non-overlapping confidence intervals denote a significant difference between samples.

### Comparative analyses among host species and across sites, in seedlings and adults

Nonmetric Multidimensional Scaling (NMDS) analyses were conducted to visualize the trends and groupings of the fungal endophytes, at individual host tree level and developmental stage, using Bray-Curtis distance for quantitative (abundance) data. NMDS was run under a random starting configuration using the metaMDS function in Vegan R version 2.4–2^64^. To obtain a global solution (minimum global stress) and avoid termination of convergence upon recovery of two minimum stress solutions (local minimum), a minimum (50) and maximum (1,000) number of iterations were set according to metaMDS developer recommendations. Analysis of Similarity (ANOSIM) was used to evaluate the effects of host species and host developmental stage on endophytic communities^65^. ANOSIM was run using the Vegan R version 2.4–2. The ANOSIM statistic (R) is a measure of the similarity between groups. R values range between 1 and 0, where R = 1 indicates the groups are completely dissimilar, and R = 0 indicates the groups are completely similar. The significance of (R) was determined with permutation tests using 999 replicates^66^. For compatibility with NMDS, Bray-Curtis distance was used to calculate ANOSIM^67^.

Fungal endophyte community rate of distance decay was calculated according to Nikola and White^68^, the assumption being that community similarities decrease with increasing geographical distance. Endophytic turnover patterns within site (local range 0.00–5.00 km) and across sites (regional range 0.00–200 km) were examined by constructing two distance matrices, one based on the presence/absence of an OTU (Jaccard), and the other based on geographic distances between samples (Euclidean). Geographic distance between individual tree hosts, within and across collection sites, was determined by first converting their geographic coordinates into Cartesian points and then calculating the Euclidean distance between them. The distance decay relationship was calculated as the slope of a least-squares linear regression on the geographic distance and the fungal endophyte community similarity, based on Jaccard index. In addition, we tested whether the slope of the distance decay curve of each collection site was significantly different from zero using a randomization with 1,000 iterations^69^.

The distribution of fungal richness for each site, tree species and age group was visualized as a Venn diagram using the VIB-UGENT Venn Diagram Tool^70^. Species abundances were ranked for each site, tree species and age group using the Rankabundance function in BiodiversityR version 2.8–0. Fungal taxa were considered core (abundant) species if they occurred in more than 50% of the individuals within a tree species within a site^71^. Putative species were parsed into six ecological guilds based on genus level using the FunGuild database^72^ including: (1) entomopathogenic, (2) fungicolous, (3) plant, (4) saprotroph, (5) wood decay, or 6) undetermined for taxa with no established ecological life history strategy. FunGuild is the current standard for use when assigning ecological function groups for consistency^73–76^. For the fungal species in dominant orders, assignment of function was confirmed based on an extensive review of literature.

### Fine scale comparative analyses between adults and seedlings, using culture-independent approaches

For three host species in NAPO (HEGU, HENI, and MISP), both culture-based and culture-independent sampling techniques were applied, and endophytic diversity, richness and distribution were examined as described above. Sequence reads (number of reads per OTU) and species incidence frequency (number of samples from which an OTU was captured) were analysed for the culture-independent dataset.

## Supplementary information

Supplemental Information

## Data Availability

Culture-dependent DNA sequences: Genbank accessions MH267812 - MH268167; Culture-independent DNA sequences: Genbank accessions MK761391 - MK762527.
