# Supplementary material for: Exploration of stem endophytic communities revealed developmental stage as one of the drivers of fungal endophytic community assemblages in two Amazonian hardwood genera
Source: Sci Rep. 2019 Sep 3;9:12685. doi: 10.1038/s41598-019-48943-2 (PMC6722055; doi:10.1038/s41598-019-48943-2)

Exploration of stem endophytic communities revealed developmental stage as one of the drivers of fungal endophytic community assemblages in two Amazonian hardwood genera

Demetra N. Skaltsas*[1,2], Fernanda Badotti[3], Aline Bruna Martins Vaz[4], Felipe Ferreira da Silva[4], Romina Gazis[5], Kenneth Wurdack[6], Lisa Castlebury[7], Aristóteles Góes-Neto[3], Priscila Chaverri[1,8]

[1]University of Maryland, Department of Plant Science and Landscape Architecture, 2112 Plant Sciences Building, College Park, Maryland 20742, USA

[2]U.S. Department of Agriculture, Agricultural Research Service, Mycology and Nematology Genetic Diversity and Biology Laboratory, 10300 Baltimore Avenue, Beltsville, Maryland 20705, USA; Oak Ridge Institute for Science and Education, ARS Research Participation Program, MC-100-44, Oak Ridge, TN 37831, USA

[3]Centro Federal de Educação Tecnológica de Minas Gerais, Departamento de Química, 30421-169, Belo Horizonte, Minas Gerais 30421-169, Brazil

[4]Universidade Federal de Minas Gerais, Departamento de Microbiologia, 31270-901, Belo Horizonte, Minas Gerais 31270-901, Brazil

[5]University of Florida, Department of Plant Pathology, Tropical Research & Education Center, 18905 SW 280 Street, Homestead, Florida 33031, USA

[6]Smithsonian Institution, Department of Botany, National Museum of Natural History, P.O. Box 37012, Washington, District of Columbia 20013, USA

[7]U.S. Department of Agriculture, Agricultural Research Service, Mycology and Nematology Genetic Diversity and Biology Laboratory, 10300 Baltimore Avenue, Beltsville, Maryland 20705, USA

[8]Escuela de Biología, Centro de Investigaciones en Productos Naturales, Universidad de Costa Rica, San Pedro, San José 11501, Costa Rica

Corresponding author: Demetra N. Skaltsas, email: demetraskaltsas@gmail.com.

U.S. Department of Agriculture, Agricultural Research Service, Mycology and Nematology Genetic Diversity and Biology Laboratory, 10300 Baltimore Avenue, Beltsville, Maryland 20705, USA

Phone: +1 240 481 9380

Tables

Supplementary Table S1 Representative isolates obtained in this study, with their corresponding GenBank accession numbers and function guild assignment.

OTU No.	Taxonomic assignment	Representative isolate	GenBank no.	Function guild assignment
1	*Absidia species 1*	PNB02_2A	MH267813	Saprotroph
2	*Absidia species 2*	PNB13_7C1	MH267812	Saprotroph
3	*Abundisporus species 1*	AMB17_3A	MH267814	Wood decay
4	*Acaromyces species 1*	GPB23_8B	MH267815	Entomopathogenic
5	*Acremoniopsis species 1*	PNB22_1A	MH267816	Saprotroph
6	*Acremonium species 1*	PNB07_7A3	MH267819	Fungicolous
7	*Acremonium species 2*	JMB07_1A1	MH267817	Fungicolous
8	*Acremonium species 3*	PXB21_6B	MH267818	Fungicolous
9	*Acrocalymma species 1*	AHGB04_6B	MH267820	Fungicolous
10	*Acrodictys species 1*	AHGB14_8B	MH267821	Saprotroph
11	*Agaricomycetes species 1*	PNB22_4A	MH267822	Undetermined
12	*Albonectria species 1*	PXB17_6B	MH267823	Plant pathogen
13	*Alloconiothyrium species 1*	AMB12_8A	MH267824	Saprotroph
14	*Alternaria species 1*	AHB25_4A	MH267825	Plant pathogen
15	*Annulohypoxylon species 1*	AHB05_1B	MH267826	Wood decay
16	*Annulohypoxylon species 2*	JMB08_7A1	MH267827	Wood decay
17	*Ascomycota species 1*	JMGB06_5A1	MH267829	Undetermined
18	*Ascomycota species 2*	PXB02_1B	MH267830	Undetermined
19	*Ascomycota species 3*	PXB20_6B	MH267831	Undetermined
20	*Aspergillus species 1*	AHB20_9B	MH267835	Saprotroph
21	*Aspergillus species 2*	JHB13_8C	MH267832	Saprotroph
22	*Aspergillus species 3*	AHB20_4B	MH267840	Saprotroph
23	*Aspergillus species 4*	JMB13_2BC	MH267837	Saprotroph
24	*Aspergillus species 5*	JHB08_1A	MH267833	Saprotroph
25	*Aspergillus species 6*	PXB15_2A	MH267836	Saprotroph
26	*Aspergillus species 7*	AHB20_3A	MH267839	Saprotroph
27	*Aspergillus species 8*	PXB18_7A	MH267834	Saprotroph

OTU No.	Taxonomic assignment	Representative isolate	GenBank no.	Function guild assignment
28	*Aspergillus species 9*	AHB10_3A	MH267838	Saprotroph
29	*Beltrania species 1*	GPB03_3B	MH267841	Saprotroph
30	*Beltraniella species 1*	AHGB21_9A1	MH267843	Saprotroph
31	*Beltraniella species 2*	JHGB09_6A	MH267842	Saprotroph
32	*Biatriospora species 2*	PXB15_7A	MH267844	Fungicolous
33	*Bionectriaceae species 1*	AHB01_5B	MH267846	Undetermined
34	*Bionectriaceae species 2*	PXB07_1A	MH267845	Undetermined
35	*Bjerkander species 1*	PXB08_5B	MH267847	Wood decay
36	*Boliniales species 2*	AMB21_6A	MH267848	Undetermined
37	*Botryosphaeriales species 1*	JHGB09_1A	MH267849	Undetermined
38	*Calonectria species 1*	JMGB02_4A	MH267850	Plant pathogen
39	*Castanediella species 1*	JHGB10_4A	MH267851	Saprotroph
40	*Castanediella species 2*	JHGB24_6A	MH267852	Saprotroph
41	*Ceriporia species 1*	JHB23_4C1	MH267854	Wood decay
42	*Ceriporia species 2*	AHB01_4A	MH267853	Wood decay
43	*Chaetosphaeriaceae 1*	AHGB16_2A	MH267855	Undetermined
44	*Chloridium species 1*	PXB03_2A	MH267856	Wood decay
45	*Chrysoporthe species 1*	JHGB18_6A	MH267857	Plant pathogen
46	*Cladosporium species 1*	JHB02_4D	MH267859	Plant pathogen
47	*Cladosporium species 2*	JHGB02_6B1A1	MH267858	Plant pathogen
48	*Cladosporium species 3*	JHGB21_1A	MH267861	Plant pathogen
49	*Cladosporium species 4*	AHB17_5A	MH267860	Plant pathogen
50	*Clavicipitaceae species 1*	PNB11_6A	MH267862	Entomopathogenic
51	*Clonostachys species 1*	JMGB02_4B	MH267864	Saprotroph
52	*Clonostachys species 2*	GPB10_3B	MH267866	Saprotroph
53	*Clonostachys species 3*	AHB27_2B	MH267865	Saprotroph
54	*Clonostachys species 4*	PNB12_2A	MH267863	Saprotroph
55	*Colletotrichum species 1*	AMGB14_1B	MH267868	Plant pathogen
56	*Colletotrichum species 10*	AHGB10_3A	MH267871	Plant pathogen
57	*Colletotrichum species 11*	GPB14_2B	MH267872	Plant pathogen
58	*Colletotrichum species 12*	GPB23_1A	MH267879	Plant pathogen

OTU No.	Taxonomic assignment	Representative isolate	GenBank no.	Function guild assignment
59	*Colletotrichum species 13*	AMGB09_6B	MH267873	Plant pathogen
60	*Colletotrichum species 14*	AHGB15_8A	MH267881	Plant pathogen
61	*Colletotrichum species 15*	AHGB04_2B	MH267870	Plant pathogen
62	*Colletotrichum species 16*	AHGB04_8C	MH267880	Plant pathogen
63	*Colletotrichum species 17*	PNB12_3C2	MH267876	Plant pathogen
64	*Colletotrichum species 2*	AMGB17_5A1	MH267869	Plant pathogen
65	*Colletotrichum species 3*	AHGB10_8A	MH267882	Plant pathogen
66	*Colletotrichum species 4*	AMGB19_6A	MH267875	Plant pathogen
67	*Colletotrichum species 5*	AHGB02_7A	MH267874	Plant pathogen
68	*Colletotrichum species 6*	AHGB10_5A	MH267878	Plant pathogen
69	*Colletotrichum species 7*	AHGB19_4B	MH267883	Plant pathogen
70	*Colletotrichum species 8*	AHGB10_5C	MH267867	Plant pathogen
71	*Colletotrichum species 9*	AHGB03_5A	MH267877	Plant pathogen
72	*Conferticium species 1*	AMB14_7B	MH267884	Wood decay
73	*Conoideocrella species 1*	AHB21_4B2	MH267885	Entomopathogenic
74	*Cophinforma species 1*	JHGB09_5A	MH267886	Saprotroph
75	*Coprinellus species 1*	PXB01_5B	MH267887	Saprotroph
76	*Cordyceps species 1*	AHB01_1A	MH267888	Entomopathogenic
77	*Coriolopsis species 1*	AHB04_2A	MH267889	Saprotroph
78	*Corticiales species 1*	JHGB12_7B	MH267890	Undetermined
79	*Corynespora species 1*	AHGB22_6A2	MH267891	Plant pathogen
80	*Cosmospora species 1*	AMB03_3A	MH267892	Fungicolous
81	*Cryphonectriaceae species 1*	JHGB17_5B	MH267893	Plant pathogen
82	*Curvularia species 1*	GPB32_9B	MH267894	Plant pathogen
83	*Cylindrium species 1*	PNB29_2B	MH267895	Saprotroph
84	*Cyphellophora species 1*	AHGB32_3B	MH267896	Saprotroph
85	*Daedalea species 1*	JHGB24_6B	MH267897	Plant pathogen
86	*Daldinia species 1*	PXB05_4A	MH267898	Saprotroph
87	*Debaryomyces species 1*	AHB19_9A	MH267899	Saprotroph
88	*Dentocorticium species 1*	JHGB17_2A	MH267900	Saprotroph
89	*Diaporthales species 1*	PXB20_7A	MH267901	Undetermined

OTU No.	Taxonomic assignment	Representative isolate	GenBank no.	Function guild assignment
90	*Diaporthales species 2*	JHGB13_7A	MH267902	Undetermined
91	*Diaporthe species 1*	JMGB10_5B1	MH267918	Plant pathogen
92	*Diaporthe species 10*	AHGB26_4A	MH267913	Plant pathogen
93	*Diaporthe species 11*	AHGB02_2A	MH267915	Plant pathogen
94	*Diaporthe species 12*	AHGB02_1A	MH267914	Plant pathogen
95	*Diaporthe species 13*	AHGB25_8B	MH267903	Plant pathogen
96	*Diaporthe species 14*	AHGB04_3A	MH267919	Plant pathogen
97	*Diaporthe species 15*	AHB22_6B	MH267908	Plant pathogen
98	*Diaporthe species 16*	AHGB05_1B	MH267909	Plant pathogen
99	*Diaporthe species 17*	GXB18_7A	MH267906	Plant pathogen
100	*Diaporthe species 18*	JHGB06_5A1	MH267920	Plant pathogen
101	*Diaporthe species 2*	GXB11_4A	MH267916	Plant pathogen
102	*Diaporthe species 3*	GPB21_9A	MH267905	Plant pathogen
103	*Diaporthe species 4*	AHGB01_1B	MH267912	Plant pathogen
104	*Diaporthe species 5*	JMGB13_6A	MH267917	Plant pathogen
105	*Diaporthe species 6*	GXB21_9A	MH267904	Plant pathogen
106	*Diaporthe species 7*	JMGB14_3A2	MH267910	Plant pathogen
107	*Diaporthe species 8*	GPB18_1A	MH267907	Plant pathogen
108	*Diaporthe species 9*	JMGB04_6B	MH267911	Plant pathogen
109	*Dothideomycetes species 1*	AHGB13_8A	MH267923	Undetermined
110	*Dothideomycetes species 2*	AHB03_4A	MH267924	Undetermined
111	*Dothideomycetes species 3*	JMB08_8A	MH267927	Undetermined
112	*Dothideomycetes species 4*	PNB05_9B	MH267922	Undetermined
113	*Dothideomycetes species 5*	AMB12_5B	MH267928	Undetermined
114	*Dothideomycetes species 6*	AMB23_9B	MH267925	Undetermined
115	*Dothideomycetes species 7*	JHGB05_3A	MH267926	Undetermined
116	*Dothideomycetes species 8*	AHGB03_6B	MH267921	Undetermined
117	*Endomelanconiopsis species 1*	PXB02_3B	MH267929	Saprotroph
118	*Endomelanconiopsis species 2*	JHGB09_1B	MH267931	Saprotroph
119	*Endomelanconiopsis species 3*	AMB09_5A	MH267930	Saprotroph
120	*Engyodontium species 1*	AMB04_2B2	MH267932	Entomopathogenic

OTU No.	Taxonomic assignment	Representative isolate	GenBank no.	Function guild assignment
121	*Entonaema species 1*	AHB18_5B	MH267934	Saprotroph
122	*Entonaema species 2*	JHGB08_1A	MH267933	Saprotroph
123	*Eucasphaeria species 1*	AHB04_5B	MH267935	Saprotroph
124	*Eutypella species 1*	AHGB29_3B	MH267937	Plant pathogen
125	*Eutypella species 2*	JHGB22_6A2	MH267938	Plant pathogen
126	*Eutypella species 3*	PNB18_5A	MH267936	Plant pathogen
127	*Exophiala species 1*	JHB07_7C	MH267939	Saprotroph
128	*Fellomyces species 1*	PNB12_6A	MH267940	Fungicolous
129	*Fusarium species 2*	GPB13_6A2	MH267946	Plant pathogen
130	*Fusarium species 3*	PNB33_2B	MH267942	Plant pathogen
131	*Fusarium species 4*	JMGB04_3A	MH267943	Plant pathogen
132	*Fusarium species 5*	JMGB14_7B	MH267944	Plant pathogen
133	*Fusarium species 6*	GPB25_1B	MH267941	Plant pathogen
134	*Fusarium species 7*	AMB05_2A	MH267945	Plant pathogen
135	*Fusicolla species 1*	PNB01_2A	MH267947	Saprotroph
136	*Ganoderma species 1*	PNB15_5B1	MH267949	Plant pathogen
137	*Ganoderma species 2*	PNB22_7	MH267948	Plant pathogen
138	*Gliomastix species 1*	PXB15_8A	MH267952	Saprotroph
139	*Gliomastix species 2*	AMB07_3A	MH267950	Saprotroph
140	*Gliomastix species 3*	AHB04_1B	MH267951	Saprotroph
141	*Gloeophyllum species 1*	AHGB09_1B1	MH267953	Wood decay
142	*Gongronella species 1*	AMB06_6A	MH267954	Saprotroph
143	*Graphium species 1*	PNB14_9B	MH267956	Saprotroph
144	*Graphium species 2*	AHB20_2A	MH267955	Saprotroph
145	*Gymnopilus species 1*	JHGB16_6A	MH267957	Wood decay
146	*Helotiales species 1*	AHGB12_4B2	MH267960	Undetermined
147	*Helotiales species 2*	JMB06_2C	MH267961	Undetermined
148	*Helotiales species 3*	JMB02_5B	MH267959	Undetermined
149	*Helotiales species 4*	PXB21_8A	MH267958	Undetermined
150	*Herpotrichiellaceae species 1*	AMB17_2A	MH267963	Undetermined
151	*Herpotrichiellaceae species 2*	JHB02_9B2	MH267962	Saprotroph

OTU No.	Taxonomic assignment	Representative isolate	GenBank no.	Function guild assignment
152	*Hortaea species 1*	AHB01_2A3	MH267964	Undetermined
153	*Hyalocladosporiella species 1*	AMB23_2A	MH267965	Plant pathogen
154	*Hyaloscypha species 1*	JHB05_5B	MH267966	Wood decay
155	*Hymenochaete species 1*	PXB08_5A	MH267967	Wood decay
156	*Hyphodontia species 1*	PNB27_5A	MH267968	Wood decay
157	*Hypocreaceae species 1*	AMB03_4A	MH267969	Undetermined
158	*Hypocreales species 1*	PXB10_3B	MH267970	Undetermined
159	*Hypocreales species 2*	JHB13_6B	MH267973	Undetermined
160	*Hypocreales species 4*	AHB02_1A	MH267972	Undetermined
161	*Hypocreales species 5*	PXB19_7B	MH267971	Undetermined
162	*Hypoxylon species 1*	PNB11_8C	MH267974	Saprotroph
163	*Infundibulomyces species 1*	JHGB24_7A	MH267975	Saprotroph
164	*Irpex species 1*	JMGB02_5A	MH267976	Wood decay
165	*Isaria species 1*	PXB03_9A	MH267977	Entomopathogenic
166	*Jattaea species 1*	AMB05_9A2A	MH267978	Wood decay
167	*Kirschsteiniothelia species 1*	AHGB13_8B	MH267979	Saprotroph
168	*Kirschsteiniothelia species 2*	PNB24_8B	MH267980	Saprotroph
169	*Kretzschmaria species 1*	PNB08_9C1	MH267981	Wood decay
170	*Lachnum species 1*	JHB02_3A2	MH267982	Saprotroph
171	*Lasiodiplodia species 1*	PXB05_3A	MH267984	Plant pathogen
172	*Lasiodiplodia species 2*	AMB14_1A	MH267983	Plant pathogen
173	*Lecanicillium species 1*	JMB06_8B	MH267985	Entomopathogenic
174	*Leptodiscella species 1*	PNB23_8C	MH267986	Saprotroph
175	*Letendraea species 1*	PNB05_3A	MH267987	Saprotroph
176	*Lophiostoma species 1*	PNB31_2A	MH267988	Saprotroph
177	*Lophiostoma species 2*	PXB22_4C2	MH267989	Saprotroph
178	*Magnaporthaceae species 1*	AHGB19_4BC	MH267991	Undetermined
179	*Magnaporthaceae species 2*	AHGB25_2A	MH267990	Undetermined
180	*Marasmiellus species 1*	JHGB06_2A	MH267992	Plant pathogen
181	*Metapochonia species 1*	JHB10_4A	MH267994	Entomopathogenic
182	*Metapochonia species 2*	AHB21_6A	MH267993	Entomopathogenic

OTU No.	Taxonomic assignment	Representative isolate	GenBank no.	Function guild assignment
183	*Mucorales species 1*	PXB11_6A	MH267995	Undetermined
184	*Mucorales species 2*	PNB07_6A	MH267996	Undetermined
185	*Muscodor species 1*	JMGB05_7B	MH267998	Fungicolous
186	*Muscodor species 2*	AMGB23_6A	MH267997	Fungicolous
187	*Muscodor species 3*	AHGB10_2B	MH267999	Fungicolous
188	*Mycena species 1*	AHB15_9A1	MH268000	Saprotroph
189	*Mycoleptodiscus species 1*	JHGB12_8A	MH268001	Plant pathogen
190	*Myrothecium species 1*	AHGB03_4A	MH268002	Plant pathogen
191	*Nectria species 1*	AHB02_4A	MH268003	Plant pathogen
192	*Nectriaceae species 1*	AHB12_1C	MH268004	Undetermined
193	*Nemania species 1*	PXB16_9B	MH268005	Saprotroph
194	*Nemania species 2*	JHGB07_5B	MH268006	Saprotroph
195	*Nemania species 3*	JMGB03_2A	MH268007	Saprotroph
196	*Nemania species 5*	AMGB24_8A	MH268008	Saprotroph
197	*Nemania species 6*	AHGB23_4A	MH268009	Saprotroph
198	*Neocosmospora species 1*	AMB09_3B	MH268010	Fungicolous
199	*Neopestalotiopsis species 1*	PNB24_5A	MH268011	Plant pathogen
200	*Neopestalotiopsis species 2*	JHB09_8A	MH268012	Plant pathogen
201	*Neopestalotiopsis species 3*	AHB12_8B	MH268013	Plant pathogen
202	*Oidiodendron species 1*	JMB08_5A	MH268014	Saprotroph
203	*Oxydothis species 1*	JHGB17_3A	MH268015	Saprotroph
204	*Paecilomyces species 1*	PNB19_2A1	MH268016	Entomopathogenic
205	*Paraconiothyrium species 1*	PXB09_4A	MH268017	Fungicolous
206	*Paraconiothyrium species 2*	JMB01_2B	MH268019	Fungicolous
207	*Paraconiothyrium species 3*	PNB10_6A	MH268020	Fungicolous
208	*Paraconiothyrium species 4*	PNB15_1B1	MH268018	Fungicolous
209	*Paramyrothecium species 1*	AHGB09_6A1	MH268021	Plant pathogen
210	*Paraphaeosphaeria species 1*	AHB05_2A	MH268022	Saprotroph
211	*Penicillium species 1*	PNB11_7A	MH268031	Saprotroph
212	*Penicillium species 10*	AMB16_2A	MH268035	Saprotroph
213	*Penicillium species 11*	AHB02_6B	MH268023	Saprotroph

OTU No.	Taxonomic assignment	Representative isolate	GenBank no.	Function guild assignment
214	*Penicillium species 12*	JHB22_8A	MH268024	Saprotroph
215	*Penicillium species 13*	PNB19_6A1	MH268037	Saprotroph
216	*Penicillium species 14*	PNB05_1B	MH268038	Saprotroph
217	*Penicillium species 15*	PNB11_5B	MH268033	Saprotroph
218	*Penicillium species 16*	JHB06_3D1	MH268040	Saprotroph
219	*Penicillium species 17*	PNB13_9A	MH268026	Saprotroph
220	*Penicillium species 18*	PNB01_1B	MH268028	Saprotroph
221	*Penicillium species 2*	PNB18_6A1	MH268025	Saprotroph
222	*Penicillium species 3*	PNB11_4A	MH268030	Saprotroph
223	*Penicillium species 4*	PNB24_1B	MH268029	Saprotroph
224	*Penicillium species 5*	AMB04_1A	MH268034	Saprotroph
225	*Penicillium species 6*	PXB17_9B	MH268032	Saprotroph
226	*Penicillium species 7*	AHB27_1AB	MH268036	Saprotroph
227	*Penicillium species 8*	PXB06_8A	MH268039	Saprotroph
228	*Penicillium species 9*	PXB13_3A	MH268027	Saprotroph
229	*Peniophora species 1*	AMB03_1A	MH268042	Wood decay
230	*Peniophora species 2*	GPB11_7D	MH268041	Wood decay
231	*Pestalotiopsis species 1*	JMB08_3B2	MH268043	Plant pathogen
232	*Pezicula species 1*	PXB14_1B	MH268045	Plant pathogen
233	*Pezicula species 2*	AHGB23_2A2	MH268046	Plant pathogen
234	*Pezicula species 3*	AMGB06_5B	MH268044	Plant pathogen
235	*Phaeoacremonium species 1*	AMB18_8B2	MH268047	Plant pathogen
236	*Phaeoacremonium species 2*	AHB24_3A	MH268048	Plant pathogen
237	*Phaeophlebiopsis species 1*	JHGB25_4A	MH268049	Saprotroph
238	*Phanerochaete species 1*	PXB08_9B	MH268050	Wood decay
239	*Phanerochaete species 2*	AMB21_5A	MH268051	Wood decay
240	*Phialea species 1*	AMB18_3A	MH268052	Saprotroph
241	*Phialemoniopsis species 1*	PNB33_3A	MH268053	Saprotroph
242	*Phialemoniopsis species 2*	PXB05_5A	MH268054	Saprotroph
243	*Phialocephala species 1*	AHB14_9A1A	MH268055	Saprotroph
244	*Phialocephala species 2*	AMB14_7A	MH268056	Saprotroph

OTU No.	Taxonomic assignment	Representative isolate	GenBank no.	Function guild assignment
245	*Phlebia species 1*	AMB20_4A	MH268057	Wood decay
246	*Phlebiopsis species 1*	JHGB12_6B	MH268059	Saprotroph
247	*Phlebiopsis species 2*	JHB09_6B	MH268058	Saprotroph
248	*Phyllosticta species 1*	AHGB01_6A	MH268060	Plant pathogen
249	*Phyllosticta species 2*	AHGB12_5A	MH268062	Plant pathogen
250	*Phyllosticta species 3*	JHGB13_8A	MH268061	Plant pathogen
251	*Physisporinus species 1*	AHB01_7B	MH268063	Wood decay
252	*Pleosporales species 1*	JMGB08_3A1	MH268065	Undetermined
253	*Pleosporales species 2*	JMB15_9B	MH268067	Undetermined
254	*Pleosporales species 3*	AMB08_2B	MH268066	Undetermined
255	*Pleosporales species 4*	AHB18_9B	MH268068	Undetermined
256	*Pleosporales species 5*	AHB11_2A1A	MH268064	Undetermined
257	*Pleurostoma species 1*	AMB01_6A	MH268069	Saprotroph
258	*Polyporales species 1*	PXB17_2A	MH268071	Undetermined
259	*Polyporales species 2*	PNB28_5A	MH268070	Undetermined
260	*Pseudallescheria species 1*	JMB10_5B	MH268072	Saprotroph
261	*Pseudofusicoccum species 1*	AHGB12_2B	MH268073	Plant pathogen
262	*Pseudopestalotiopsis species 1*	GPB03_6B	MH268074	Plant pathogen
263	*Psilocybe species 1*	AHB13_3A1	MH268075	Saprotroph
264	*Purpureocillium species 1*	PXB04_1B	MH268076	Entomopathogenic
265	*Purpureocillium species 2*	AHB10_9A	MH268077	Entomopathogenic
266	*Pyrenochaetopsis species 1*	AHB10_7A	MH268078	Saprotroph
267	*Rigidoporus species 1*	AHGB17_3B	MH268079	Wood decay
268	*Roussoella species 1*	PNB08_6A	MH268080	Saprotroph
269	*Thyridariaceae species 1*	PNB16_5A1	MH268117	Saprotroph
270	*Thyridariaceae species 2*	PXB15_4B	MH268115	Saprotroph
271	*Thyridariaceae species 3*	AHB24_2B	MH268116	Saprotroph
272	*Sarocladium species 1*	AHB01_8B	MH268081	Saprotroph
273	*Schizophyllum species 1*	AHGB05_6A	MH268082	Wood decay
274	*Scopuloides species 1*	PNB08_8A2	MH268083	Wood decay
275	*Scytalidium species 1*	PXB08_9A	MH268084	Fungicolous

OTU No.	Taxonomic assignment	Representative isolate	GenBank no.	Function guild assignment
276	*Scytalidium species 2*	AMB15_1A	MH268086	Fungicolous
277	*Scytalidium species 3*	JHB14_8A	MH268087	Fungicolous
278	*Scytalidium species 4*	PNB27_4C_1	MH268085	Fungicolous
279	*Septobasidium species 1*	PNB30_8B	MH268088	Entomopathogenic
280	*Sistotrema species 1*	AHGB05_5B	MH268090	Mycorrhizal
281	*Sistotrema species 2*	PNB21_8	MH268089	Mycorrhizal
282	*Sistotremastrum species 1*	AHB20_7A2	MH268091	Saprotroph
283	*Sordariales species 1*	JHGB25_5B	MH268093	Undetermined
284	*Sordariales species 2*	JMGB03_6B1	MH268092	Undetermined
285	*Sordariomycetes species 1*	JHGB15_7B	MH268098	Undetermined
286	*Sordariomycetes species 10*	JHB04_2A	MH268099	Undetermined
287	*Sordariomycetes species 2*	JHGB16_1A	MH268095	Undetermined
288	*Sordariomycetes species 3*	JHGB02_5B	MH268096	Undetermined
289	*Sordariomycetes species 5*	JHGB10_2A	MH268094	Undetermined
290	*Sordariomycetes species 6*	JHGB06_3B	MH268097	Undetermined
291	*Sphaeronaemella species 1*	PXB04_2B	MH268102	Saprotroph
292	*Stachybotryaceae species 1*	AHB27_4F	MH268103	Undetermined
293	*Stereum species 1*	AMB05_6B	MH268105	Saprotroph
294	*Stereum species 2*	JHGB02_3A	MH268104	Saprotroph
295	*Talaromyces species 1*	PNB11_5A1	MH268107	Saprotroph
296	*Talaromyces species 2*	PNB17_5A2	MH268108	Saprotroph
297	*Talaromyces species 3*	PXB20_3A	MH268106	Saprotroph
298	*Talaromyces species 4*	AHB30_8A	MH268112	Saprotroph
299	*Talaromyces species 5*	JHB10_8A	MH268110	Saprotroph
300	*Talaromyces species 6*	PXB08_8A	MH268109	Saprotroph
301	*Talaromyces species 7*	AHB27_4G	MH268111	Saprotroph
302	*Thozetella species 1*	JMGB15_5A	MH268114	Saprotroph
303	*Thozetella species 2*	JMGB06_7A1	MH268113	Saprotroph
304	*Tinctoporellus species 1*	PNB27_6A	MH268119	Wood decay
305	*Tinctoporellus species 2*	JHGB01_1A	MH268118	Wood decay
306	*Tolypocladium species 1*	PNB01_1A1	MH268121	Entomopathogenic

OTU No.	Taxonomic assignment	Representative isolate	GenBank no.	Function guild assignment
307	*Tolypocladium species 2*	AHB21_5B	MH268123	Entomopathogenic
308	*Tolypocladium species 3*	PNB17_7A1	MH268124	Entomopathogenic
309	*Tolypocladium species 4*	AHB13_7A	MH268122	Entomopathogenic
310	*Tolypocladium species 5*	JHB08_3B	MH268120	Entomopathogenic
311	*Trametes species 1*	AHB23_1A	MH268125	Wood decay
312	*Trematosphaeriaceae species 1*	AHGB26_2B	MH268126	Undetermined
313	*Tremellales species 1*	PNB09_9A	MH268127	Undetermined
314	*Trichocomaceae species 1*	PNB28_4A	MH268128	Saprotroph
315	*Trichoderma species 1*	AMB08_8B	MH268131	Fungicolous
316	*Trichoderma species 10*	PXB02_8B	MH268140	Fungicolous
317	*Trichoderma species 11*	AHB02_2A	MH268151	Fungicolous
318	*Trichoderma species 12*	AHB14_3B	MH268132	Fungicolous
319	*Trichoderma species 13*	PNB09_5A	MH268139	Fungicolous
320	*Trichoderma species 15*	PXB01_8B	MH268142	Fungicolous
321	*Trichoderma species 16*	PNB03_2A	MH268138	Fungicolous
322	*Trichoderma species 17*	PXB09_9B	MH268135	Fungicolous
323	*Trichoderma species 18*	AMB08_2A	MH268136	Fungicolous
324	*Trichoderma species 19*	PNB14_8A	MH268149	Fungicolous
325	*Trichoderma species 2*	AHB15_1A	MH268143	Fungicolous
326	*Trichoderma species 20*	AMB22_3A1A	MH268133	Fungicolous
327	*Trichoderma species 21*	AHB03_3A	MH268148	Fungicolous
328	*Trichoderma species 22*	PXB06_1A	MH268137	Fungicolous
329	*Trichoderma species 23*	AHB05_3B2	MH268134	Fungicolous
330	*Trichoderma species 24*	AMB11_7A	MH268130	Fungicolous
331	*Trichoderma species 3*	PNB10_7A2	MH268150	Fungicolous
332	*Trichoderma species 4*	PNB15_6B	MH268147	Fungicolous
333	*Trichoderma species 5*	JHB08_2A	MH268144	Fungicolous
334	*Trichoderma species 6*	PNB12_1A1	MH268141	Fungicolous
335	*Trichoderma species 7*	AMB17_6A	MH268145	Fungicolous
336	*Trichoderma species 8*	JMB03_8B	MH268146	Fungicolous
337	*Trichoderma species 9*	AHB24_4B	MH268129	Fungicolous

OTU No.	Taxonomic assignment	Representative isolate	GenBank no.	Function guild assignment
338	*Umbelopsis species 1*	AHB27_3F2	MH268152	Saprotroph
339	*Verticillium species 1*	PXB21_4A	MH268153	Nematicidal
340	*Virgaria species 1*	PNB13_4A1	MH268154	Saprotroph
341	*Wardomyces species 1*	AHB16_5A	MH268155	Saprotroph
342	*Xenoacremonium species 1*	AHB27_3B	MH268156	Saprotroph
343	*Xylaria species 1*	JMGB05_9B	MH268157	Saprotroph
344	*Xylaria species 4*	GPB18_4A	MH268158	Saprotroph
345	*Xylaria species 5*	PXB08_4A	MH268162	Saprotroph
346	*Xylaria species 6*	JHGB20_9	MH268161	Saprotroph
347	*Xylaria species 7*	AHGB04_1A	MH268159	Saprotroph
348	*Xylaria species 8*	GPB06_4C	MH268160	Saprotroph
349	*Xylariaceae species 1*	PXB19_5A	MH268163	Saprotroph
350	*Anungitea species 1*	JHGB05_3B	MH267828	Undetermined
351	*Xylariales species 2*	PNB02_6D	MH268164	Undetermined
352	*Sordariomycetes species 4*	PNB10_2A	MH268100	Undetermined
353	*Sordariomycetes species 7*	JHGB23_7B	MH268101	Undetermined
354	*Xylomelasma species 1*	AMB02_6A	MH268165	Undetermined
355	*Xylona species 1*	PNB16_8B	MH268166	Undetermined
356	*Zasmidium species 1*	AHB32_8A	MH268167	Saprotroph

Supplementary Table S2 Culture-dependent method: Observed and estimated species richness (SR) and diversity (Shannon Hill numbers and Simpson Hill numbers), including upper (UCL) and lower (LCL) confidence intervals, of endophytes per location (Amazon Conservatory for Tropical Studies Biological Station [NAPO], Allpahuayo-Mishana National Reserve [ALPE], and Jenaro Herrera Research Center [JEHE]), tree species and tree developmental stage. Non-overlapping confidence levels signify a significant difference in richness or diversity, while partially overlapping confidence levels do not guarantee non-significance (Chao et al. 2014).

Site	Tree species	Dev. stage	Observed							Estimated								
			SR	Shannon	LCL	UCL	Simpson	LCL	UCL	SR	LCL	UCL	Shannon	LCL	UCL	Simpson	LCL	UCL
ALPE	*H. guianensis*	Adult	39	29	20	38	20	12	27	168	77	480	70	29	113	27	20	44
		Seedling	43	15	11	19	6	4	8	91	59	183	19	15	25	6	6	8
	H. pauciflora	Adult	44	32	26	38	22	15	30	132	74	301	66	33	100	31	22	45
		Seedling	39	16	11	21	8	6	10	206	80	715	27	16	39	9	8	12
	M. elata	Adult	53	39	30	49	26	19	33	138	89	256	82	54	110	36	26	52
		Seedling	23	17	11	23	13	9	17	119	39	597	33	17	53	16	13	23
	M. spruceana	Adult	32	22	14	31	15	10	21	102	53	259	53	22	90	21	15	35
		Seedling	19	8	6	11	4	2	6	43	25	118	12	8	18	4	4	6
JEHE	*H. brasiliensis*	Adult	28	23	18	28	19	15	24	46	33	87	38	27	49	30	19	43
		Seedling	57	38	30	47	23	15	31	124	84	225	64	45	83	28	23	39
	H. nitida	Adult	17	13	7	18	9	4	15	40	23	114	25	13	42	13	9	24
		Seedling	40	25	20	30	15	10	20	62	48	101	36	27	45	17	15	24
	M. spruceana	Adult	39	23	14	32	12	6	18	197	86	572	58	23	103	14	12	22
		Seedling	45	19	15	23	9	7	11	56	49	80	23	19	28	9	9	12
NAPO	*H. guianensis*	Adult	90	55	45	64	32	25	38	218	150	359	93	72	114	37	32	48
		Seedling	35	12	10	15	6	5	7	51	40	89	14	12	17	6	6	8
	H. nitida	Adult	43	35	25	46	26	14	37	170	88	400	117	38	197	46	26	84
		Seedling	19	10	7	14	7	5	9	49	25	162	15	10	22	7	7	10
	M. spruceana	Adult	79	67	55	79	54	42	66	292	175	550	192	119	265	100	63	136
		Seedling	18	6	4	7	3	2	4	26	20	60	6	6	8	3	3	4

Supplementary Table S3 Analysis of similarities (ANOSIM) results of the fungal endophyte communities for tree developmental stages (seedling, adult) and host species for both culture-dependent and culture-independent methods using Bray-Curtis distance for quantitative (abundance) data. Each sampling location (Amazon Conservatory for Tropical Studies Biological Station [NAPO], Allpahuayo-Mishana National Reserve [ALPE], and Jenaro Herrera Research Center [JEHE]) was analyzed independently and pooled together (All locations). Significant test results (P <0.005) are in bold.

Method	Site	Host level	ANOSIM	
			R	p
**Culture-dependent**	**ALPE**	Developmental stage	0.752	**0.001**
		Tree Species	0.062	0.014
	**JEHE**	Developmental stage	0.780	**0.001**
		Tree Species	0.030	0.093
	**NAPO**	Developmental stage	0.761	**0.001**
		Tree Species	0.033	0.071
	**ALL locations**	Developmental stage	0.729	**0.001**
		Tree species	0.017	0.093
**Culture-independent**	**NAPO Incidence frequency**	Developmental stage	0.919	**0.001**
		Tree species	0.027	0.103
	**NAPO Sequence Reads**	Developmental stage	0.789	**0.001**
		Tree Species	0.026	0.114

Supplementary Table S4 The rate of distance decay (DD) of the fungal endophyte communities for each tree host across and within sampling locations (Amazon Conservatory for Tropical Studies Biological Station [NAPO], Allpahuayo-Mishana National Reserve [ALPE], and Jenaro Herrera Research Center [JEHE]). P values (P <0.005) in bold indicate a significant increase in community dissimilarity with increasing geographic distance.

Location	Developmental stage	Tree species	Geographic distance range (km)	Slope Intercept	Rate of DD	P value
ALPE	Adult tree	*Hevea guianensis*	0.00 - 2.54	0.978	-0.042	0.129
		Hevea pauciflora	0.00 - 2.53	0.947	-0.009	0.25
		Micrandra elata	0.00 - 1.25	0.95	-0.001	0.965
		Micrandra spruceana	0.00 - 1.23	1.015	-0.078	0.017
	Seedling tree	*Hevea guianensis*	0.00 - 2.54	0.797	-0.014	0.399
		Hevea pauciflora	0.00 - 2.53	0.786	0.013	0.208
		Micrandra elata	0.00 - 1.25	0.906	-0.004	0.899
		Micrandra spruceana	0.00 - 1.23	0.612	0.054	0.179
NAPO	Adult tree	*Hevea guianensis*	0.00 - 3.24	0.959	-0.01	0.097
		Hevea nitida	0.00 - 4.87	0.946	0.003	0.699
		Micrandra spruceana	0.00 - 2.81	0.984	-0.002	0.563
	Seedling tree	*Hevea guianensis*	0.00 - 3.24	0.705	-0.033	0.249
		Hevea nitida	0.00 - 4.87	0.681	0.06	**0.001**
		Micrandra spruceana	0.00 - 2.81	0.612	0.001	0.952
JEHE	Adult tree	*Hevea brasiliensis*	0.00 - 4.06	0.989	-0.034	0.01
		Hevea nitida	0.00 - 4.02	0.993	-0.011	0.31
		Micrandra spruceana	0.00 - 3.76	0.896	0.008	0.42
	Seedling tree	*Hevea brasiliensis*	0.00 - 4.06	0.9	-0.004	0.563
		Hevea nitida	0.00 - 4.02	0.898	0.01	0.366
		Micrandra spruceana	0.00 - 3.76	0.788	-0.001	0.926
All locations	Adult tree	*Hevea guianensis*	0.00 - 97.65	0.965	-0.00054	**0.001**
		Hevea nitida	0.00 - 200.3	0.982	-0.00017	0.078
		Micrandra spruceana	0.00 - 198.2	0.973	-0.00031	**0.001**
	Seedling tree	*Hevea guianensis*	0.00 - 97.65	0.758	-0.00048	0.011
		Hevea nitida	0.00 - 200.3	0.914	-0.00046	**0.004**
		Micrandra spruceana	0.00 - 198.2	0.814	-0.00134	**0.001**

Supplementary Table S5 The number and percent of endophyte species overlap within and across developmental stages (seedling, adult), per tree species, method (culture-dependent and culture independent), and location (Amazon Conservatory for Tropical Studies Biological Station [NAPO], Allpahuayo-Mishana National Reserve [ALPE], and Jenaro Herrera Research Center [JEHE]). Percentages of shared endophytes were calculated with singletons included and excluded from the datasets.

Site	Tree host dev. stage	Tree host species	No. endophyte species	No. singletons	No. shared endo species within dev. stage	No. shared endo species across dev. stage	% endophyte overlap within dev. stages		% endophyte overlap across dev. stages	
							with singletons	without singletons	with singletons	without singletons
ALPE	Adult	*H. guianensis*	39	16	21	4	54%	91%	10%	17%
		H. pauciflora	44	21	21	2	48%	91%	5%	9%
		M. elata	53	26	22	1	41%	79%	2%	4%
		M. spruceana	32	14	17	2	53%	94%	6%	12%
	Seedling	*H. guianensis*	43	14	25	4	58%	86%	9%	14%
		H. pauciflora	39	16	23	2	59%	100%	5%	9%
		M. elata	23	7	15	1	65%	94%	4%	6%
		M. spruceana	19	3	15	2	79%	94%	11%	13%
JEHE	Adult	*H.*	28	12	12	3	43%	75%	11%	19%
		H. nitida	17	8	8	2	47%	89%	12%	22%
		M. spruceana	39	24	12	3	31%	80%	8%	20%
	Seedling	*H.*	57	25	31	3	54%	97%	5%	9%
		H. nitida	40	12	25	2	63%	89%	5%	7%
		M. spruceana	45	9	28	3	62%	78%	7%	8%
NAPO	Adult	*H. guianensis*	90	40	38	6	42%	76%	7%	12%
		H. nitida	43	16	26	4	60%	96%	9%	15%
		M. spruceana	79	47	27	3	34%	84%	4%	9%
	Seedling	*H. guianensis*	35	11	15	6	43%	63%	17%	25%
		H. nitida	19	7	11	4	58%	92%	21%	33%
		M. spruceana	18	3	14	3	78%	93%	17%	20%
NAPO Culture Independent	Adult	*H. guianensis*	317	225	31	3	10%	34%	1%	3%
		H. nitida	133	108	20	3	15%	80%	9%	15%
		M. spruceana	122	87	23	2	19%	66%	4%	9%
	Seedling	*H. guianensis*	72	32	35	3	49%	88%	4%	8%
		H. nitida	53	33	20	3	38%	100%	21%	33%
		M. spruceana	562	430	30	2	5%	23%	17%	20%

Supplementary Table S6 The distribution of core species per location (Amazon Conservatory for Tropical Studies Biological Station [NAPO], Allpahuayo-Mishana National Reserve [ALPE], and Jenaro Herrera Research Center [JEHE]), separated by developmental stage, and tree species. Columns denote a core species and an "X" indicates that the endophyte species was identified as a core species within a particular location, dataset and tree host. Core species: *Annulohypoxylon* species 1 (Annul 1), *Colletotrichum* species 11 (Colle 11), *Diaporthe* species 1 (Diapo 1), *Diaporthe* species 14 (Diapo 14), *Diaporthe* species 19 (Diapo 19), *Diaporthe* species 4 (Diapo 4), *Diaporthe* species 5 (Diapo 5), *Diaporthe* species 7 (Diapo 7), *Diaporthe* species 8 (Diapo 8), *Diaporthe* species 9 (Diapo 9), *Neopestalotiopsis* species 3 (Neope 3), *Pezicula* species 1 (Pezic 1), *Trichoderma* species 11 (Trich 11), and *Trichoderma* species 21 (Trich 21).

**Site**	**Tree host species**	Adult tree endophyte core species					**Seedling tree endophyte core species**								
		Trich 21	Neope 3	Diapo 19	Annul 1	Trich 11	Diapo 4	Diapo 7	Diapo 14	Pezic 1	Diapo 5	Diapo 8	Colle 11	Diapo 1	Diapo 9
ALPE	*H. guianensis*	**x**	-	-	-	-	**x**	**x**	-	-	-	-	-	-	-
	H. pauciflora	**x**	-	-	-	-	**x**	**x**	-	-	-	-	-	-	-
	M. elata	-	**x**	-	-	-	**x**	**x**	**x**	**x**	-	-	-	-	-
	M. spruceana	-	-	-	-	-	-	-	-	**x**	-	-	-	-	-
JEHE	*H. brasiliensis*	-	-	-	-	-	-	**x**	-	-	-	-	-	**x**	-
	H. nitida	-	-	-	-	-	-	-	-	-	-	-	-	**x**	-
	M. spruceana	-	-	-	-	**x**	-	-	-	-	-	-	-	**x**	**x**
NAPO	*H. guianensis*	-	-	-	**x**	-	**x**	**x**	-	-	**x**	-	-	-	-
	H. nitida	-	-	-	-	-	-	**x**	-	-	-	-	**x**	-	-
	M. spruceana	-	-	-	-	-	-	**x**	-	-	**x**	**x**	-	-	-

Supplementary Table S7 Culture-independent method: Observed and estimated species richness (SR) and diversity (Shannon Hill numbers and Simpson Hill numbers), including upper (UCL) and lower (LCL) confidence intervals, of endophytes for Amazon Conservatory for Tropical Studies Biological Station [NAPO], tree species and tree developmental stage. Partially overlapping confidence levels do not guarantee non-significance (Chao et al. 2014).

Site	Tree species	Dev. stage	Observed							Estimated								
			SR	Shannon	LCL	UCL	Simpson	LCL	UCL	SR	LCL	UCL	Shannon	LCL	UCL	Simpson	LCL	UCL
NAPO	*H. guianensis*	Adult	318	221	199	242	134	112	156	844	674	1096	446	389	502	174	138	210
		Seedling	72	45	39	50	32	27	36	104	85	148	57	49	64	36	32	43
	H. nitida	Adult	133	105	88	122	76	56	96	660	399	1179	346	239	452	123	76	173
		Seedling	53	37	31	43	27	21	34	161	91	361	66	45	87	35	27	45
	M. spruceana	Adult	122	72	60	83	42	32	53	391	261	645	134	102	165	50	42	60
		Seedling	562	385	351	418	222	192	252	2052	1649	2602	938	816	1061	289	234	344

Supplementary Table S8 The distribution of core species recovered from tree hosts in Amazon Conservatory for Tropical Studies Biological Station (NAPO) using culture-independent method. HEGU: *Hevea guianensis*, HENI: *Hevea nitida* and MISP: *Micrandra spruceana.* Columns denote a core species and an "X" indicates that the endophyte species was identified as a core species within a particular location, dataset and tree host. Core species: *Acremonium* species 3 (Acre 3), Agaricales species 15.2 (Agar 15.2), Basidiomycota species 21 (Basta 21), *Beltrania* species 1 (Beltr 1), *Beltraniella* species 1 (Belta 1), *Beltraniella* species 5 (Bella 5), *Colletotrichum* species 1 (Colle 1), *Colletotrichum* species 2 (Colle 2), *Cyphellophora* species 1 (Cyphe 1), *Debaryomyces* species 1 (Debar 1), *Debaryomyces* species 6 (Deb 6), *Debaryomyces* species 16 (Debar 16), *Debaryomyces* species 18 (Debar 18), *Graphidaceae* species 2.2 (Grap 2.2), Hypocreales species 3 (Hyp 3), *Sarocladium* species 1 (Saro 1), *Tolypocladium* species 1 (Tolyp 1), *Tolypocladium* species 2 (Tolyp 2), Tricholomataceae 1.1 (Mycen 1.1), Tricholomataceae 1.3 (Mycen 1.3), Xylariales species 4.1 (Xylal 4.1), and *Xylodon* species 5 (Xlod 5).

**Tree species**	**Adult tree endophyte core species**							**Seedling tree endophyte core species**															
	Deb 6	Hyp 3	Toly 2	Saro 1	Toly 1	Acre 3	Agar 1	Debar 1	Debar 16	Debar 18	Grap 2	Mycen 1.3	Mycen 1.1	Colle 1	Colle 2	Xlod 5	Basta 21	Bella 5	Belta 1	Beltr 1	Cyphe 1	Debar 6	Xylal 4
HEGU	x	x	x	x	x	-	-	x	x	x	x	x	x	x	x	x	-	-	-	-	-	-	-
HENI	x	x	x	x	-	-	-	x	x	x	x	x	x	-	-	-	-	-	-	-	-	-	-
MISP	x	x	x	x	-	x	x	x	x	x	x	x	x	-	-	-	x	x	x	x	x	x	x

Supplementary Table S9 The proportion of function guilds separated by developmental stage, method (culture-dependent and culture independent), and tree host species sampled from Amazon Conservatory for Tropical Studies Biological Station (NAPO).

Developmental stage	Method	Tree species	Proportion of function guild					
			Entomopathogenic	Fungicolous	Plant pathogen	Saprotroph	Undetermined	Wood decay
Adult	Culture dependent	*H. guianensis*	7%	36%	5%	34%	9%	8%
		H. nitida	9%	19%	5%	34%	17%	16%
		M. spruceana	6%	14%	11%	42%	13%	13%
		Site total	7%	27%	7%	37%	12%	11%
	Culture independent	*H. guianensis*	19%	12%	12%	38%	19%	1%
		H. nitida	19%	10%	10%	48%	13%	1%
		M. spruceana	32%	7%	6%	32%	21%	2%
		Site total	23%	10%	10%	38%	18%	1%
Seedling	Culture dependent	*H. guianensis*	1%	1%	79%	19%	0%	1%
		H. nitida	2%	6%	77%	16%	0%	0%
		M. spruceana	0%	3%	95%	2%	1%	0%
		Site total	1%	2%	84%	12%	0.2%	0%
	Culture independent	*H. guianensis*	0%	1%	26%	39%	32%	2%
		H. nitida	0%	0%	18%	44%	34%	4%
		M. spruceana	1%	1%	9%	39%	44%	6%
		Site total	0%	1%	26%	39%	32%	2%

Supplementary Figure S1 Diversity results from culture-dependent approach from trees distributed in Amazon Conservatory for Tropical Studies Biological Station (NAPO). Species accumulation and diversity curves for endophytes sampled from (a) seedling and (b) adult trees separated by host species (HEGU: *Hevea guianensis*, HENI: *Hevea nitida* and MISP: *Micrandra spruceana*). Metrics include richness (q=0), Shannon HN (q=1), Simpson's HN (q=2).

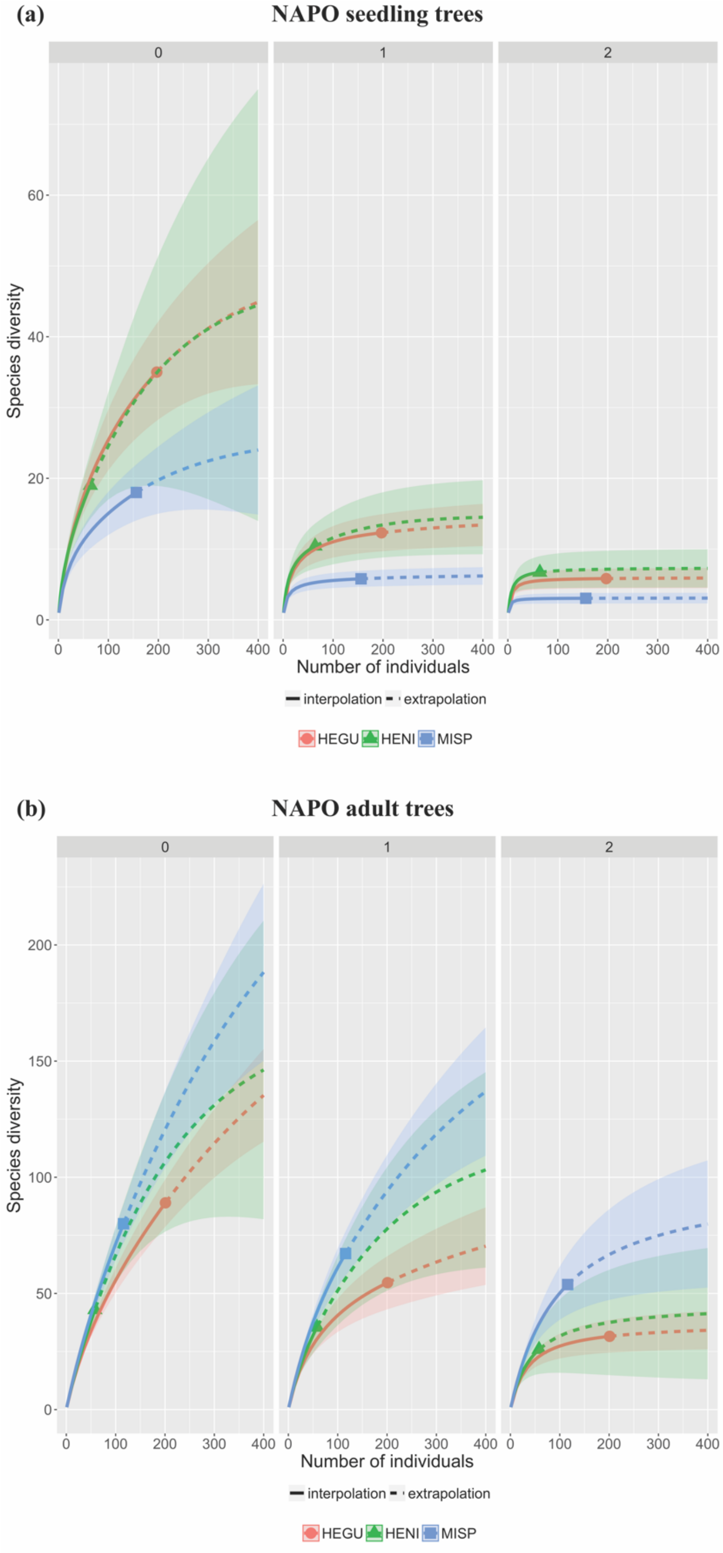

Supplementary Figure S2 Diversity results from culture-dependent approach from trees distributed in Allpahuayo-Mishana National Reserve (ALPE). Species accumulation and diversity curves for endophytes sampled from (a) seedling and (b) adult trees separated by host species (HEGU: *Hevea guianensis*, HEPA: *Hevea pauciflora*, MIEL: *Micrandra elata* and MISP: *Micrandra spruceana*). Metrics include richness (q=0), Shannon HN (q=1), Simpson's HN (q=2).

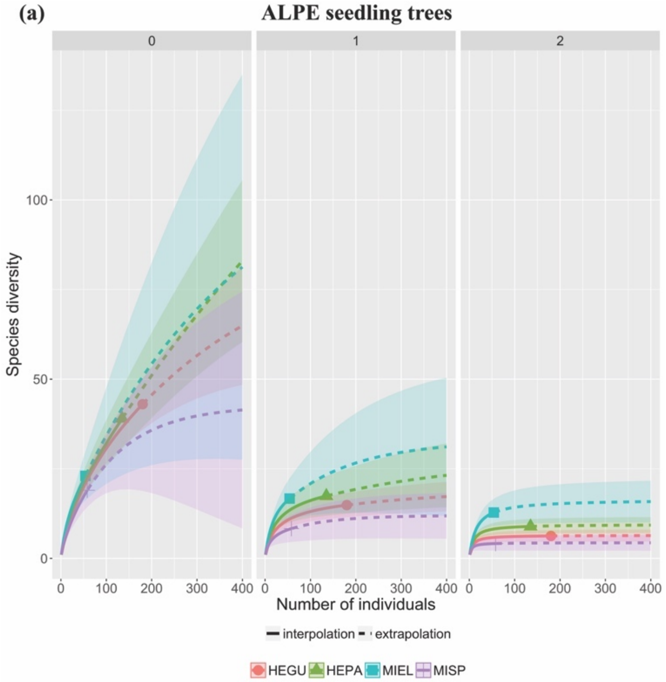

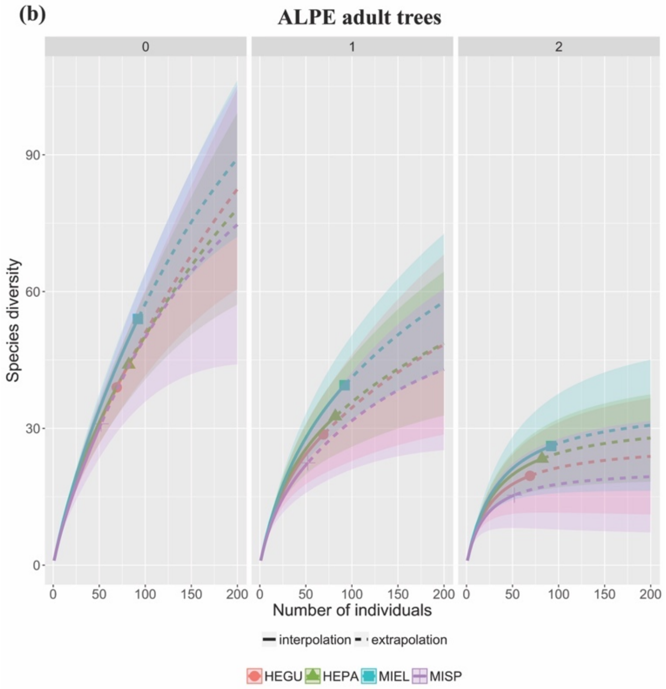

Supplementary Figure S3 Diversity results from culture-dependent approach from trees distributed in Jenaro Herrera Research Center (JEHE). Species accumulation and diversity curves for endophytes sampled from (a) seedling and (b) adult trees separated by host species (HEBR: *Hevea brasiliensis*, HENI: *Hevea nitida* and MISP: *Micrandra spruceana*). Metrics include richness (q=0), Shannon HN (q=1), Simpson's HN (q=2).

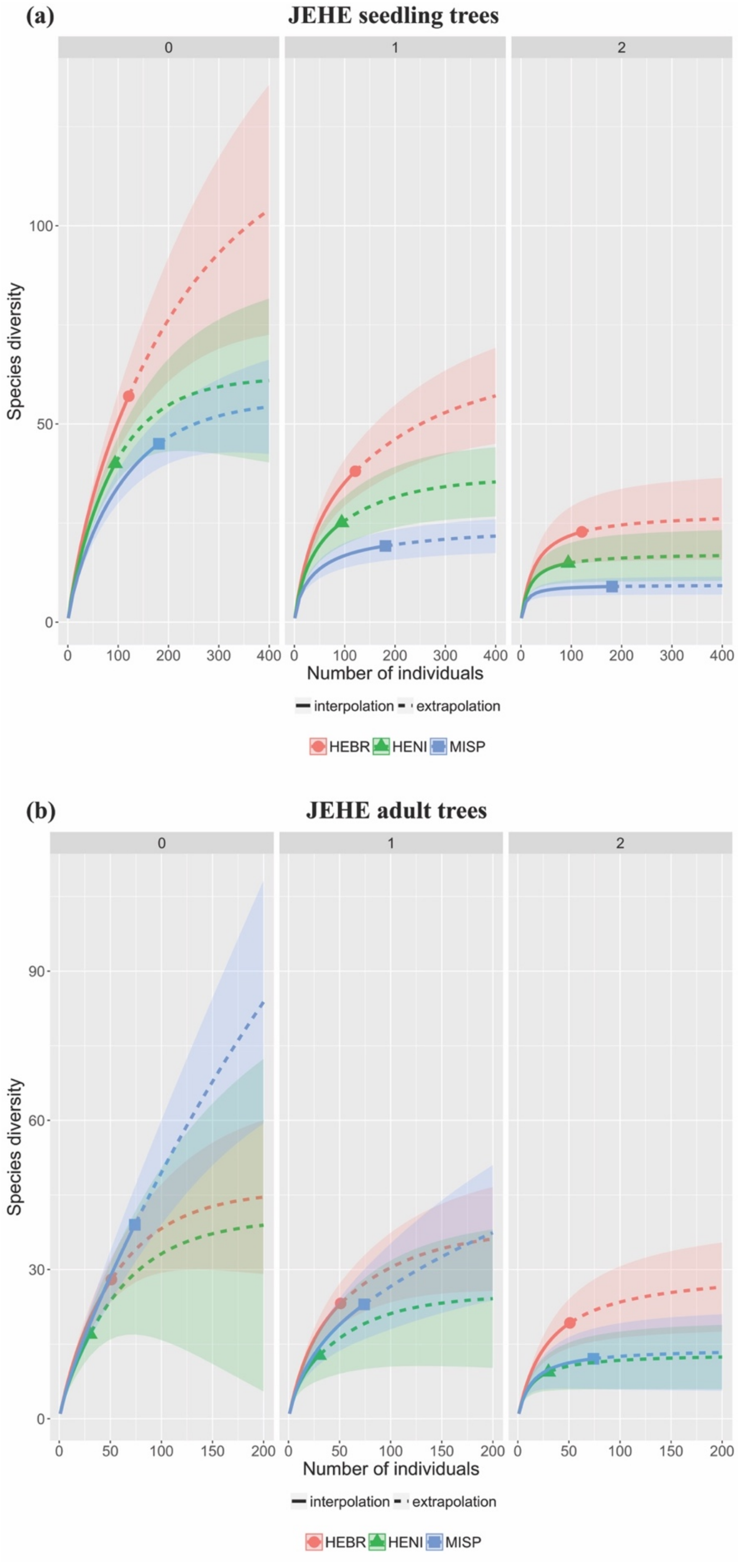

Supplementary Figure S4 Culture-dependent approach. Nonmetric Multidimensional Scaling (NMDS) analyses using Bray-Curtis distance for quantitative (abundance) with stress values for all three locations and data partitioned by (a) developmental stage (Ad: Adult, Sd: Seedling), and (b) tree species (HEBR: *Hevea brasiliensis*, HEGU: *Hevea guianensis*, HENI: *Hevea nitida*, HEPA: *Hevea pauciflora*, MIEL: *Micrandra elata*, and MISP: *Micrandra spruceana*).

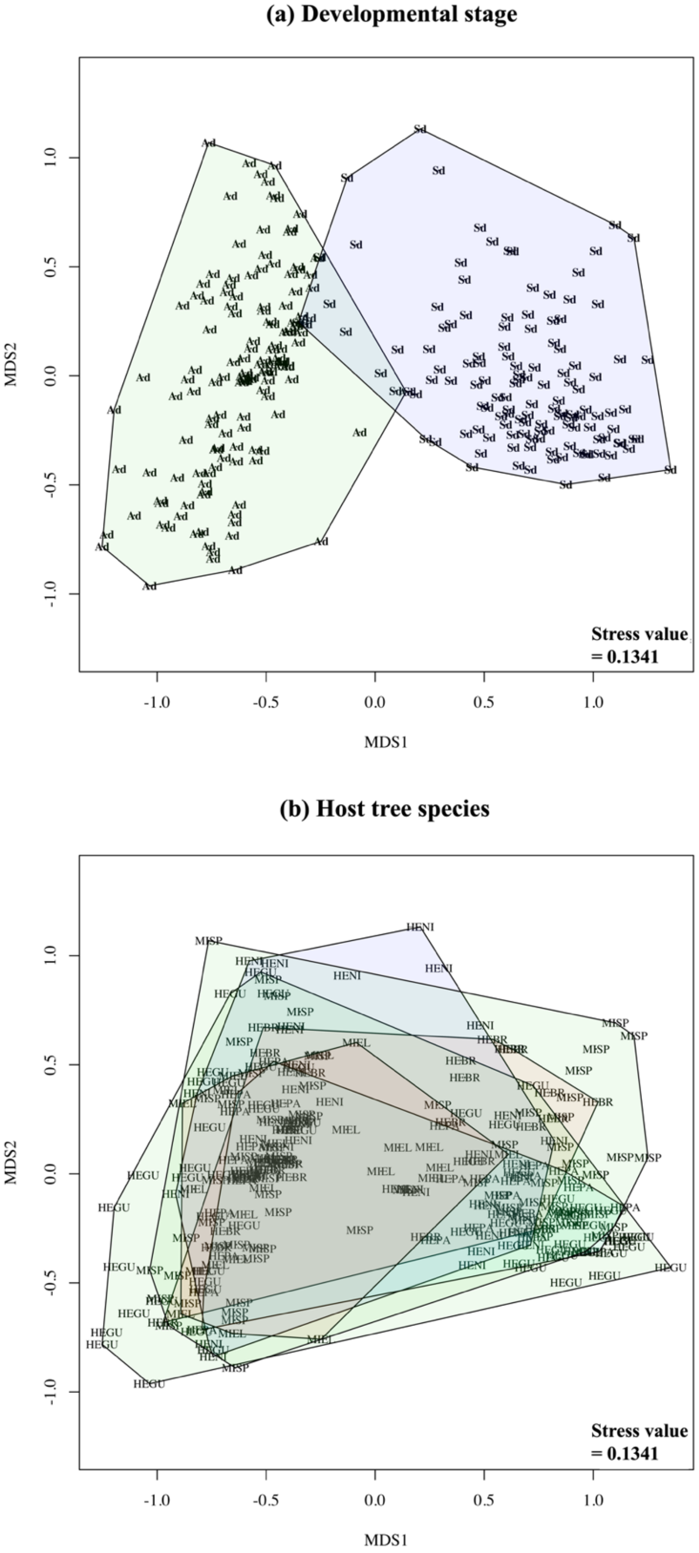

Supplementary Figure S5 Culture-dependent approach. Nonmetric Multidimensional Scaling (NMDS) analyses using Bray-Curtis distance for quantitative (abundance) data with stress values. Data partitioned by developmental stage (Ad: Adult [green], Sd: Seedling [blue]) for each tree host species (a) HEBR: *Hevea brasiliensis*, (b) HEGU: *Hevea guianensis*, (c) HENI: *Hevea nitida*, (d) HEPA: *Hevea pauciflora*, (e) MIEL: *Micrandra elata*, and (f) MISP: *Micrandra spruceana*.

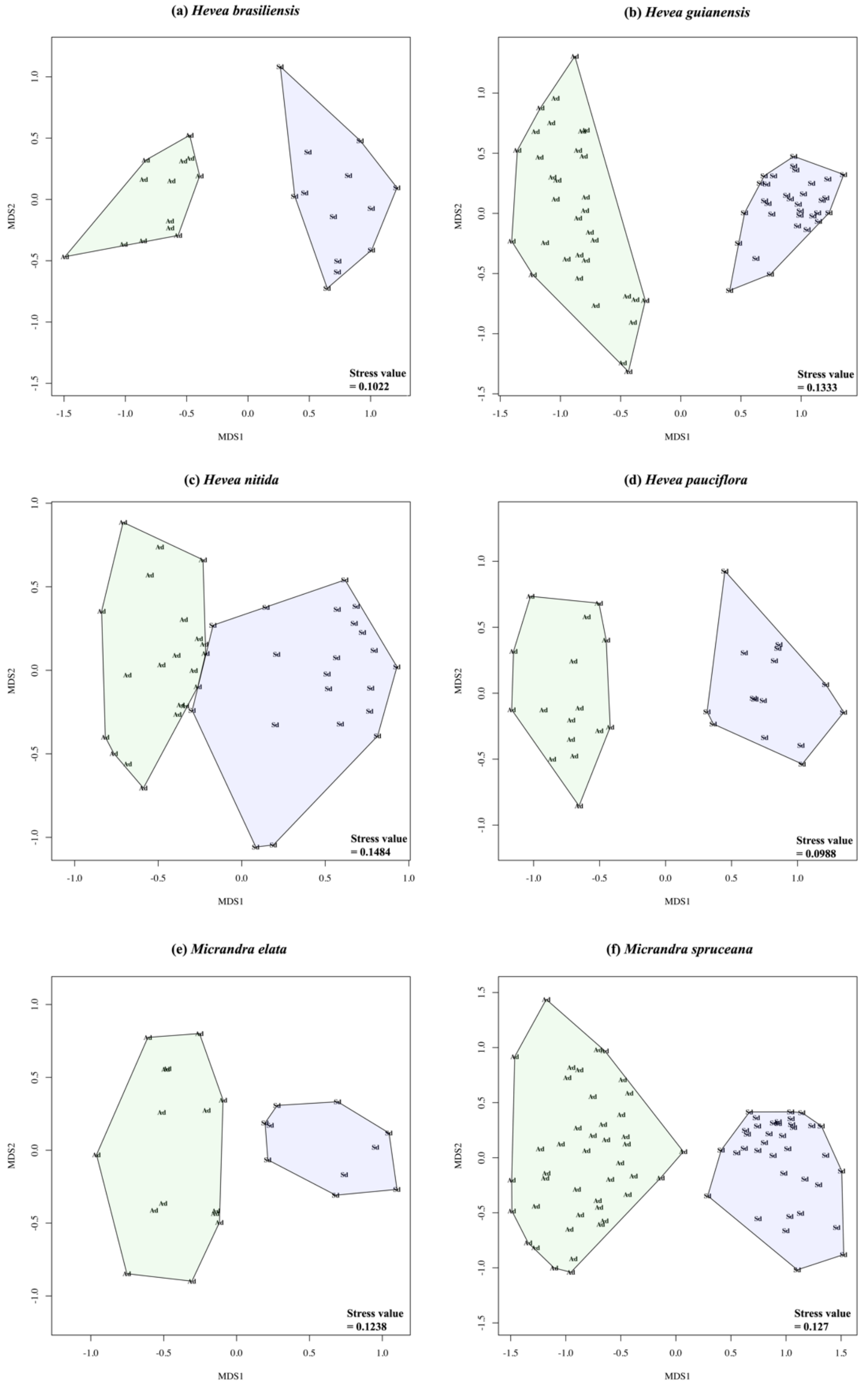

Supplementary Figure S6 Venn diagrams showing the overlap of fungal species among adult tree communities (HEBR: *Hevea brasiliensis*, HEGU: *Hevea guianensis*, HENI: *Hevea nitida*, HEPA: *Hevea pauciflora*, MIEL: *Micrandra elata*, and MISP: *Micrandra spruceana*) per location (a) the Amazon Conservatory for Tropical Studies Biological Station (NAPO), (b) Allpahuayo-Mishana National Reserve (ALPE), and (c) Jenaro Herrera Research Center (JEHE).

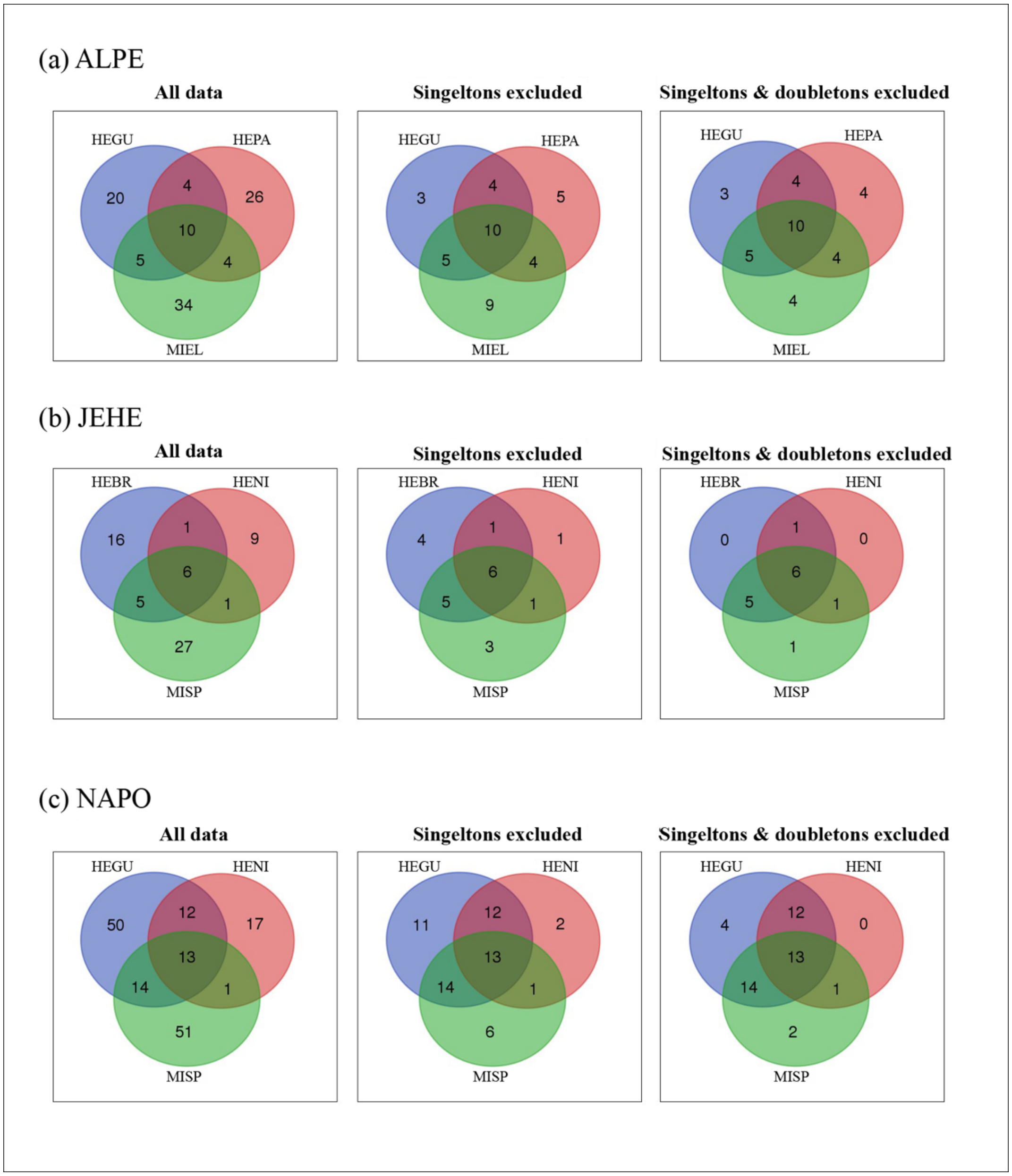

Supplementary Figure S7 Diversity results from culture-independent approach. Species accumulation and diversity curves for endophytes sampled from seedling and adult trees separated by host species. Trees were sampled from the Amazon Conservatory for Tropical Studies Biological Station (NAPO). Metrics include richness (q=0), Shannon HN (q=1), Simpson's HN (q=2).

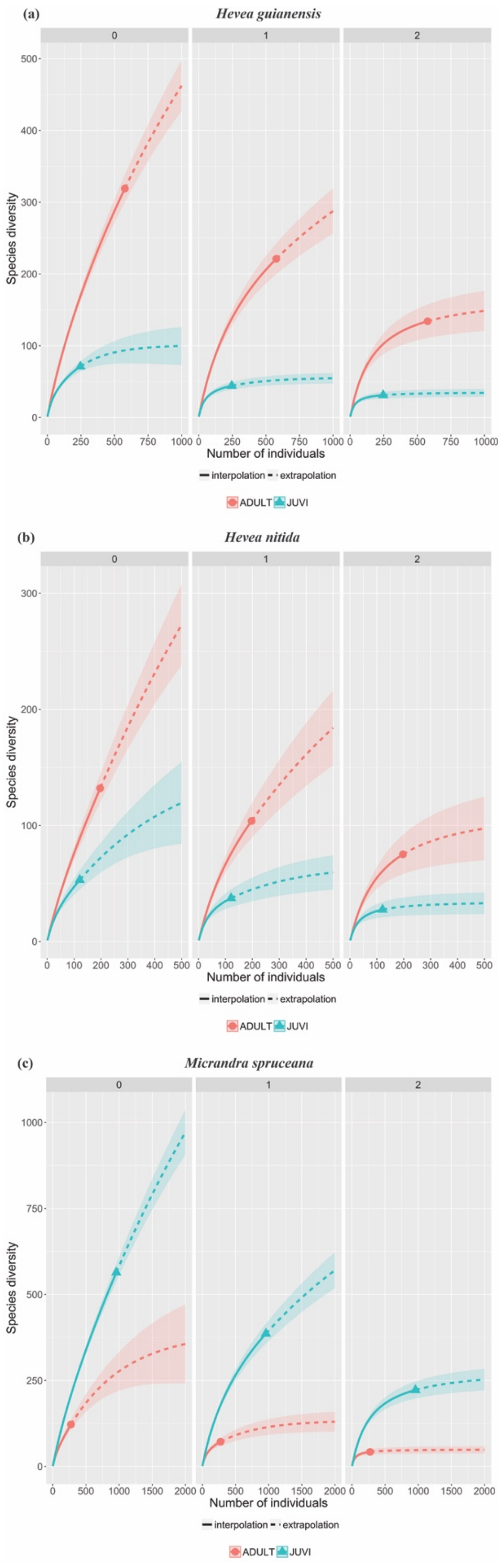

Supplementary Figure S8 Culture-independent approach diversity results. Species accumulation and diversity curves for endophytes sampled from (a) seedling and (b) adult trees separated by host species (HEGU: *Hevea guianensis*, HENI: *Hevea nitida* and MISP: *Micrandra spruceana*). Trees were sampled from the Amazon Conservatory for Tropical Studies Biological Station (NAPO). Metrics include richness (q=0), Shannon HN (q=1), Simpson's HN (q=2).

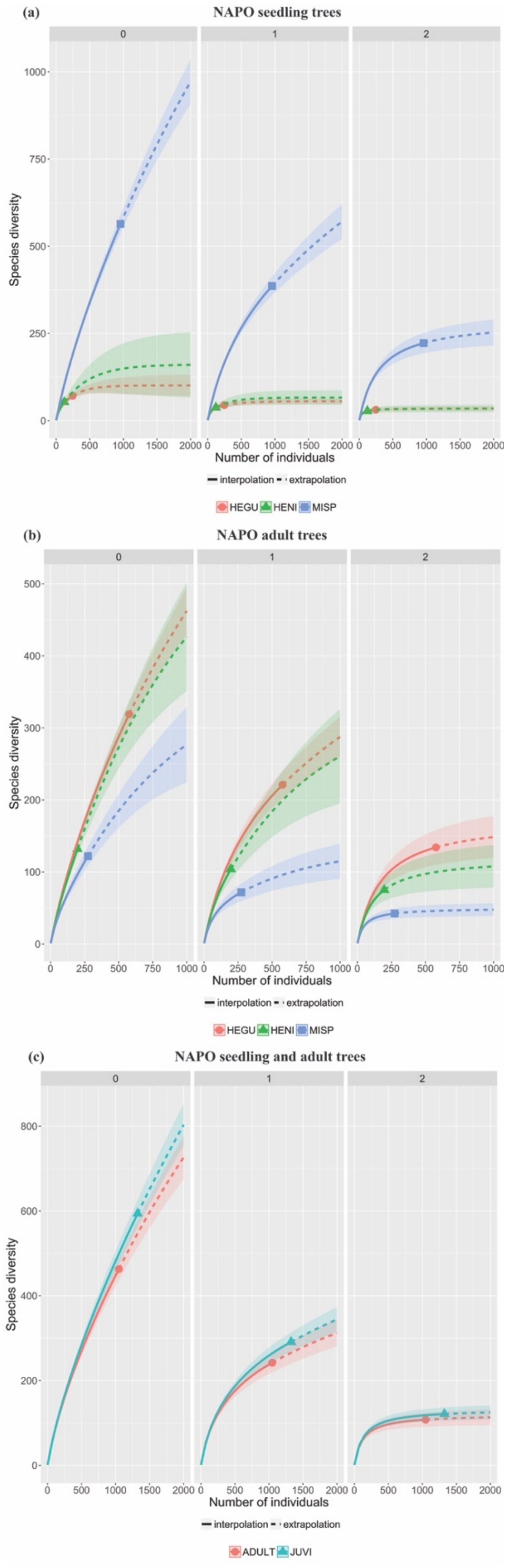

Supplement: Supplementary file 1 — Supplemental Information [file 41598_2019_48943_MOESM1_ESM.pdf]
